# Recovery from multi‐millennial natural coastal hypoxia in the Stockholm Archipelago, Baltic Sea, terminated by modern human activity

**DOI:** 10.1002/lno.11575

**Published:** 2020-08-18

**Authors:** Niels A. G. M. van Helmond, Bryan C. Lougheed, Annika Vollebregt, Francien Peterse, Guillaume Fontorbe, Daniel J. Conley, Caroline P. Slomp

**Affiliations:** ^1^ Department of Earth Sciences, Faculty of Geosciences Utrecht University Utrecht The Netherlands; ^2^ Department of Geology Lund University Lund Sweden; ^3^ Department of Earth Sciences Uppsala University Uppsala Sweden; ^4^ Laboratoire des Sciences du Climat et de l'Environnement LSCE/IPSL, CEA CNRS‐UVSQ, Université Paris‐Saclay Gif‐sur‐Yvette France

## Abstract

Enhanced nutrient input and warming have led to the development of low oxygen (hypoxia) in coastal waters globally. For many coastal areas, insight into redox conditions prior to human impact is lacking. Here, we reconstructed bottom water redox conditions and sea surface temperatures (SSTs) for the coastal Stockholm Archipelago over the past 3000 yr. Elevated sedimentary concentrations of molybdenum indicate (seasonal) hypoxia between 1000 b.c.e. and 1500 c.e. Biomarker‐based (TEX_86_) SST reconstructions indicate that the recovery from hypoxia after 1500 c.e. coincided with a period of significant cooling (∼ 2°C), while human activity in the study area, deduced from trends in sedimentary lead and existing paleobotanical and archeological records, had significantly increased. A strong increase in sedimentary lead and zinc, related to more intense human activity in the 18^th^ and 19^th^ century, and the onset of modern warming precede the return of hypoxia in the Stockholm Archipelago. We conclude that climatic cooling played an important role in the recovery from natural hypoxia after 1500 c.e., but that eutrophication and warming, related to modern human activity, led to the return of hypoxia in the 20^th^ century. Our findings imply that ongoing global warming may exacerbate hypoxia in the coastal zone of the Baltic Sea.

Dissolved oxygen concentrations are decreasing in marine settings globally, leading to hypoxia (O_2_ < 2 mg L^−1^), which is detrimental to most marine life (Diaz and Rosenberg 2008; Schmidtko et al. 2017). Oceanic oxygen depletion is attributed to two main causes, both linked to human activity: (1) enhanced nutrient input and (2) climatic warming (Breitburg et al. 2018). Higher surface water nutrient concentrations can boost marine primary productivity in nearshore areas, increasing the quantity of sinking organic matter and consequent oxygen consumption upon its remineralization (Anderson et al. 2002; Diaz and Rosenberg 2008). Climatic warming can stimulate marine primary productivity through a variety of mechanisms, but often generates complex responses (Laufkötter et al. 2015), including alteration of food web structures (Petchey et al. 1999). Warming also enhances respiration, decreases oxygen gas solubility, and promotes water column stratification, inhibiting mixing of the water column (Keeling and Garcia 2002; Paerl and Huisman 2009).

A combination of enhanced nutrient input, climatic warming, and strong natural density stratification has led to widespread oxygen depletion in the Baltic Sea over the past century (Gustafsson et al. 2002; Kabel et al. 2012; Carstensen et al. 2014). The main hypoxic area covers the generally deeper, central parts of the Baltic Sea (Baltic Proper), but considerable portions of its coastal zones are, or have been, (seasonally) hypoxic as well (Conley et al. 2011). Widespread oxygen depletion in the Baltic Sea is not limited to the modern human‐induced hypoxic interval, however. Such a depletion is well‐described for the Baltic Proper, where two previous intervals of widespread oxygen depletion, coinciding with the Holocene Thermal Maximum (HTM, ∼ 6000–2000 b.c.e.) and the Medieval Climate Anomaly (MCA, ∼ 800–1300 c.e.), were identified in sediment records (Zillén et al. 2008; Jilbert and Slomp 2013). During both intervals, climatic warming was likely a key driver of hypoxia (Kabel et al. 2012; Papadomanolaki et al. 2018). An important additional driver includes increases in salinity, allowing for the development of a stronger vertical salinity gradient during the HTM, due to a local maximum sea level transgression (Zillén et al. 2008). Furthermore, increased human activity during the MCA may have contributed to oxygen depletion, for example, through soil disturbance and enhanced nutrient release (Zillén and Conley 2010). However, in a recent study of diatom assemblages for three locations along the Swedish east coast, capturing the last 1000 yr, no evidence for increased productivity was observed before 1800 c.e. (Norbäck Ivarsson et al. 2019).

Knowledge about the history of hypoxia in the coastal zone of the Baltic Sea is relatively limited. Current evidence suggests a much wider variety of redox conditions in the coastal zone during the Holocene than observed in the Baltic Proper, likely depending on local trends in factors such as bottom water salinity, nutrient and freshwater inputs, temperature, water depth and water exchange with adjacent coastal areas. For example, post‐glacial rebound led to substantial shallowing and freshening at two previously studied sites in the coastal zone of the Baltic Sea (in southeast Sweden and the Bothnian Sea), resulting in increasingly oligotrophic and oxygenated conditions over most of the Holocene (Ning et al. 2016; Dijkstra et al. 2018). In the Finnish Archipelago Sea, sedimentological characteristics of MCA sediments indicate a period of modest, climate‐driven deoxygenation in a system that was otherwise largely oxygenated during the past 1500 yr prior to modern hypoxia (Jokinen et al. 2018). In contrast, the Little Belt, part of the Danish coastal zone, was continuously seasonally hypoxic over the past ∼ 8000 yr as a result of high natural nutrient loading (van Helmond et al. 2017).

Here, we study sedimentary records from three locations in the Stockholm Archipelago at an unprecedented temporal resolution. Extensive present‐day water column monitoring curated by the Swedish Meteorological and Hydrological Institute (SMHI; van Helmond et al. 2020) combined with the vicinity of the largest city in Scandinavia, makes the selected study area uniquely suited to address the role of humans in the development of (pre‐modern) hypoxia in the coastal zone of the Baltic Sea. In addition, in order to address the role of climate, we have generated the first detailed biomarker‐based sea surface temperature (SST) record for the coastal zone of the Baltic Sea. Our results indicate that the study area was (seasonally) hypoxic between 1000 b.c.e. and 1500 c.e. and that climatic cooling played a key role in terminating this interval.

## *Materials and methods*

### Study area

**Fig. 1:**
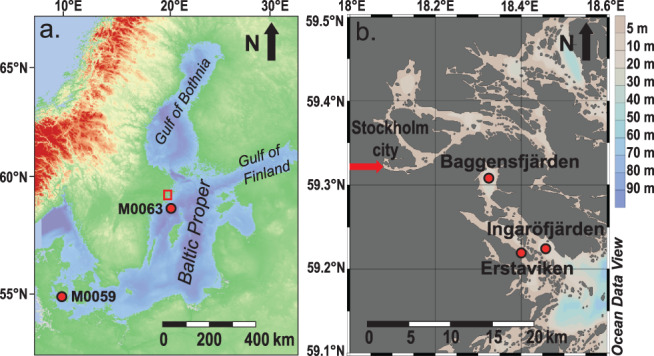
Bathymetric map of the Baltic Sea, including locations of the Baltic proper, Gulf of Bothnia, Gulf of Finland, and locations of the study sites of van Helmond et al. (2017) and Kotthoff et al. (2017) – M0059 (Little Belt, Danish Straits) and Papadomanolaki et al. (2018) – M0063 (Landsort Deep). The study area in the Stockholm Archipelago is indicated by the red box (**a**). Bathymetric map of the southwestern part of the inner and intermediate Stockholm Archipelago, with red dots to indicate the study sites in Ingaröfjärden, Erstaviken and Baggensfjärden and the red arrow indicating the outflow of the Norrström River, which connects the Stockholm Achipelago with its main freshwater source, Lake Mälaren (**b**).

The Stockholm Archipelago (Fig. 1) consists of about 30,000 mostly rocky islands surrounded by a network of basins and straits of different sizes and depths shaped by post‐glacial rebound (Hill and Wallström, 2008). Nearly freshwater conditions prevail in the most western part of the archipelago where the outflow of the Norrström River connects the Stockholm Archipelago with its main freshwater source Lake Mälaren (Fig. 1b). Eastward, salinity generally increases to ∼ 7. Large parts of the Stockholm Archipelago are or have been (seasonally) hypoxic to euxinic (no dissolved oxygen and the presence of free sulfide which is toxic to most organisms) over the past century (Jonsson 2003; Conley et al. 2011). Here, we studied sediments from three different locations in the sub‐basins Ingaröfjärden, Erstaviken and Baggensfjärden (Fig. 1b; coordinates of the different study sites are provided in Table 1), with different water depths and (vertical) water mass characteristics (Table 1).

**Table 1:** General site characteristics. mbss, meters below sea surface.

Site	Latitude (°N)	Longitude (°E)	Water depth (mbss)	Redox conditions (1996–2016)*
Ingaröfjärden	59°13′20.06″	18°27′0.18″	37.4	Oxic
Erstaviken	59°13′11.14″	18°23′40.45″	68.4	Sporadically hypoxic
Baggensfjärden	59°18′30.67″	18°19′23.45″	41.1	Seasonally hypoxic, sometimes euxinic

* Based on present‐day water column monitoring curated by the Swedish Meteorological and Hydrological Institute (SMHI; van Helmond et al. 2020).

### Sediment sampling and analysis

Gemini and piston cores (up to 68 and 600 cm sediment depth, respectively; Supplementary Fig. S1) were retrieved during a cruise with the *R/V Ocean Surveyor* in July 2015. In order to achieve the highest possible resolution and locations with continuous sedimentation, we targeted deep, sheltered parts of the three selected sub‐basins using sub‐bottom profiling to allow for optimal site selection. For Baggensfjärden, the deepest northern part of the basin was chosen in order to avoid a spoil ground, that is, an area of the seafloor reserved for the dumping of waste (e.g., dredged material), in the center of the basin. The Gemini cores were cut into 1 cm slices throughout the full length of the cores. The piston cores were cut into 1 m sections and split into working and archive halves. Sediments at all three sites consisted of generally laminated, mostly dark gray to blackish, fine‐grained silty to clayey material. Only at Baggensfjärden the lower 2 m of sediment consisted of greenish gray clay. Sediments from Erstaviken were characterized by the thickest and lowest number of laminae (Supplementary Fig. S1) suggesting the record captured the shortest period of time, and therefore was regarded most suitable for a high‐resolution study. For Ingaröfjärden and Baggensfjärden, a 1 cm slice of sediment was taken every 10 cm, while the piston core of Erstaviken was sampled from bottom to top with a resolution of 0.5 cm. Macrofossils for radiocarbon dating were picked upon sampling of the cores. Sample depths were corrected for core expansion resulting from methane‐degassing. Gemini and piston cores were spliced based on sedimentary concentrations of organic carbon (C_org_), lead (Pb), and zinc (Zn). All samples were freeze‐dried, re‐inspected for macrofossils, and powdered and homogenized using an agate mortar and pestle. Sediment porosity was determined based upon the weight loss from freeze‐drying. Details on the different sediment analyses including the full dataset are provided in the Supplementary Information.

### Organic carbon content

In brief, about 0.3 g of freeze‐dried sediment was decalcified with 1 M HCl, after which C_org_ was measured using a Fisons Instruments NA 1500 NCS analyzer at Utrecht University. The average analytical uncertainty for C_org_ was 0.06 wt.% based on duplicate analyses of sediment samples (*n* = 42).

### Total elemental composition

About 0.1 g of freeze‐dried sediment was digested in a mixture of strong acids (HF : HClO_4_ : HNO_3_, 5 : 3 : 2). After evaporating the acids, sediment residues were dissolved in 1 M HNO_3_ and measured by Inductively Coupled Plasma‐Optical Emission Spectrometry (ICP‐OES; SPECTRO ARCOS) at Utrecht University, to determine the total elemental composition. Average analytical uncertainty based on duplicate analyses of sediment samples (*n* = 103) was 1 part per million (ppm) for molybdenum (Mo), 3 ppm for Pb, 5 ppm for Zn, and 1617 ppm for aluminum (Al).

### Biomarkers

For Erstaviken, glycerol dialkyl glycerol tetraethers (GDGTs) were extracted from approximately 1 g of freeze‐dried sediment using organic solvents and isolated using small column chromatography. Relative abundances of the different GDGTs were quantified using ultra‐high‐performance liquid chromatography‐mass spectrometry (UHPLC–MS) on an Agilent 1260 infinity series instrument coupled to a 6130 quadrupole mass selective detector at Utrecht University with settings as described in (Hopmans et al. 2016). The Branched and Isoprenoid Tetraether (BIT) index (Hopmans et al. 2004), which we adjusted to include both 5‐methyl and 6‐methyl branched GDGT isomers, was calculated to determine the relative abundance of soil organic matter vs. marine organic matter. TEX_86_^L^ values were determined according to Kim et al. (2010), after which SSTs were calculated using the calibration for the Baltic Sea (Kabel et al. 2012). The average analytical uncertainty, based on duplicate measurements of polar fractions (*n* = 3), was 0.01 for TEX_86_^L^, corresponding to 0.37°C in SST.

### Core chronology

For Erstaviken, freeze‐dried sediment from the Gemini corer was measured for ^210^Pb, ^226^Ra, and ^137^Cs by direct gamma counting using a high purity germanium detector (Ortec GEM‐FX8530P4‐RB) at Lund University. The dates derived from the constant rate of supply model (Appleby and Oldfield, 1978), were, based on the best fit with the ^137^Cs profile, used for the age‐depth model for Erstaviken. In addition, ^14^C determinations on macrofossils (pine needles, leaf fragments and mollusk shells [*Macoma balthica*]) from 12 samples (11 from the piston corer and one from the Gemini corer) were carried out using a ^14^C single stage accelerated mass spectrometer (SSAMS) at Lund University. Radiocarbon reservoir age, or R(t), for mollusk macrofossils in the hydrographically dynamic Baltic Sea can vary greatly in both time and space. We therefore generated ^87^Sr/^86^Sr measurements on the mollusk shells to apply a previously developed ^87^Sr/^86^Sr‐based R(t) transfer function (Lougheed et al. 2016). At the same time, the ^87^Sr/^86^Sr‐based data were used to calculate salinity estimates following the method outlined in Ning et al. (2017) based on previous work by Widerlund and Andersson (2011). Age‐depth modeling was carried out using the *Undatable* age‐depth modeling software (Lougheed and Obrochta 2019), using 105 iterations with an xfactor of 0.1% and 20% bootstrapping enabled. The bootstrapping process enables the age‐depth modeling software to incorporate larger geochronological error in the age‐depth model for intervals where the age‐depth points display poor agreement due to, for example, age‐depth reversals.

## *Results*

### Erstaviken

**Fig. 2:**
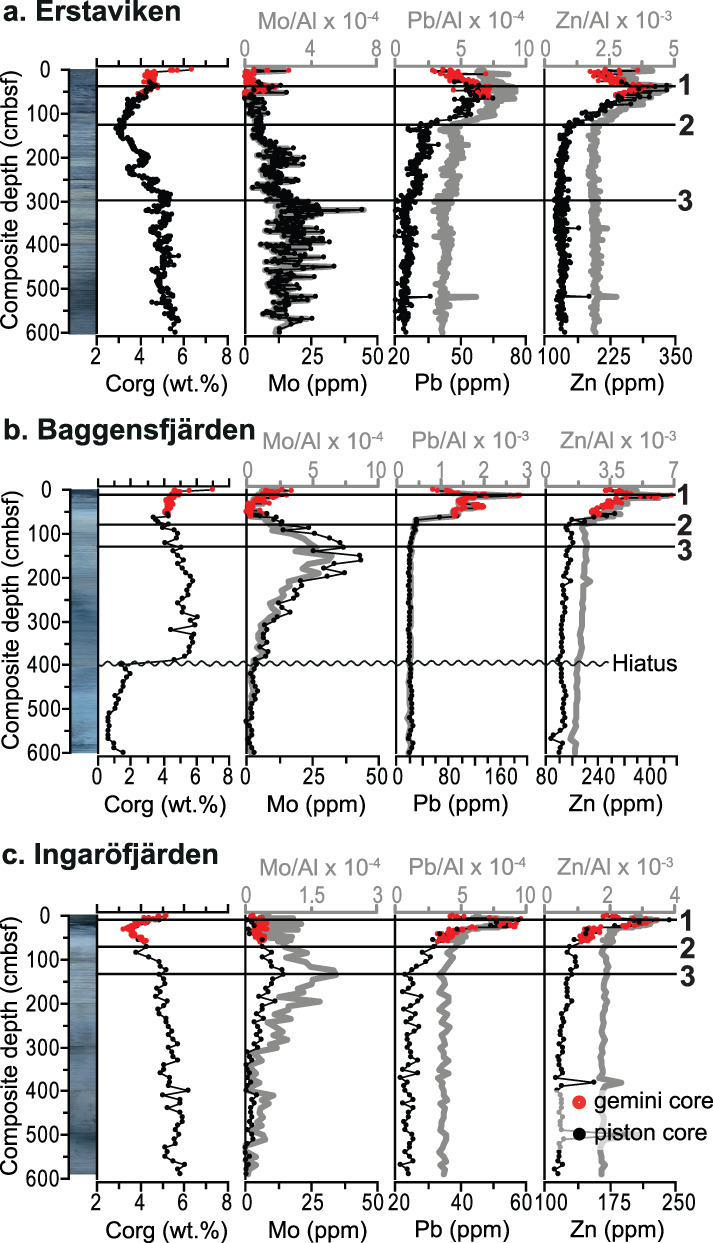
Core photographs and sedimentary concentrations of organic carbon (C_org_), molybdenum (Mo), lead (Pb), zinc (Zn), and aluminum (Al) normalized trace metal concentrations (i.e., Mo/Al, Pb/Al and Zn/Al) for the three study sites in the Stockholm Archipelago; (**a**) Erstaviken, (**b**) Baggensfjärden, and (**c**) Ingaröfjärden. Numbered horizontal black lines refer to the three identified chemostratigraphic horizons: (1) maximum modern Pb pollution; (2) onset of Zn enrichment; and (3) onset of Pb enrichment.

Sedimentary C_org_ concentrations are relatively stable (∼ 5 wt.%) from the base of the record until ∼ 280 cm below sea floor (cmbsf), after which they gradually decrease to a minimum for the record (∼ 3 wt.%) between ∼ 155 and 115 cmbsf (Fig. 2a). Subsequently, values increase again to 4.5 wt.% toward the top of the record, with an additional enrichment to ∼ 6.5 wt.% in the top 4 cm. Sedimentary Mo concentrations are above the crustal average (∼ 1–2 ppm; Turekian and Wedepohl 1961) for most of the record and particularly enriched from the base of the record until ∼ 280 cmbsf (∼ 20 ppm on average; Fig. 2a). Subsequently, Mo concentrations show a minor decrease but remain enriched (∼ 15 ppm on average) up to ∼ 160 cmbsf. Afterward, Mo concentrations drop toward average crustal values, only to show substantial enrichment again (Mo > 10 ppm) in the top 60 cm. Sedimentary Pb concentrations (Fig. 2a) show a gradual increase (from ∼ 25 to 35 ppm on average) from 300 cmbsf onward, a small decrease (to ∼ 30 ppm) between 250 till 200 cmbsf, a rapid increase from 130 to 40 cmbsf (to maximum concentrations of 64 ppm) and finally a sharp drop toward the top. Sedimentary Zn concentrations (Fig. 2a) are constant (∼ 140 ppm) for most of the record and only sharply increase from ∼ 130 to 40 cmbsf (to maximum concentrations of 335 ppm) after which they sharply drop again toward the top of the record.

### Baggensfjärden

The lower 200 cm of the record are characterized by relatively low C_org_ (< 2 wt.%; Fig. 2b). Above this interval, after a hiatus, C_org_ gradually decreases from ∼ 6 to 3.5 wt.% around 70 cmbsf. Subsequently, C_org_ increases toward 5 wt.%, while the top sediments are further enriched again. Sedimentary Mo concentrations remain around the crustal average in the lower 200 cm of the record (Fig. 2b). Subsequently, Mo concentrations gradually increase to maximum values of 43 ppm around 150 cmbsf, after which they rapidly decrease to average crustal values (55–30 cmbsf). In the top sediments, Mo concentrations increase again to values of ∼ 15 ppm. Sedimentary Pb concentrations (Fig. 2b) show a small and gradual increase from ∼ 125 cmbsf that is followed by a rapid increase from ∼ 70 cmbsf onward. Maximum concentrations of ∼ 200 ppm are reached around 15 cmbsf. Afterward, Pb concentrations decrease to ∼ 60 ppm at the top of the record. Sedimentary Zn concentrations (Fig. 2b) are constant up to ∼ 80 cmbsf (∼ 140 ppm), after which they rapidly increase to a maximum value of ∼ 470 ppm around 15 cmbsf. Subsequently, Zn concentrations decrease to 250 ppm at the top of the record.

### Ingaröfjärden

Sedimentary C_org_ gradually decreases from 6 wt.% at the base of the record to 5 wt.% around 100 cmbsf (Fig. 2c). Subsequently, C_org_ rapidly decreases to minimum values of ∼ 3 wt.% around 30 cmbsf, after which values rapidly increase again to values of 5 wt.% at the top. Sedimentary Mo concentrations are below 5 ppm in the lower 300 cm (Fig. 2c), after which they gradually increase to maximum values of 14 ppm (130–120 cmbsf). Thereafter, Mo concentrations gradually decrease again to values around 5 ppm. Sedimentary Pb concentrations (Fig. 2c) are constant (∼ 25 ppm) up to ∼ 135 cmbsf. They gradually increase to 40 ppm around 30 cmbsf, followed by a rapid increase to ∼ 60 ppm around 10 cmbsf and a decrease to ∼ 40 ppm at the top of the record. Sedimentary Zn concentrations (Fig. 2c) are constant (∼ 125 ppm) up to 70 cmbsf, after which they rapidly increase to a maximum of 240 ppm around 10 cmbsf. Subsequently, Zn concentrations decrease to ∼ 170 ppm at the top of the record.

### Trace metal normalization and chemostratigraphic correlation

For all three of the studied sites, Al‐normalized trace metal concentrations exhibit trends that are identical to the raw concentrations (Fig. 2), that is, there is a very constant detrital (background) component. At all three sites, three distinct geochemical levels are recognized that can be used to correlate the records to each other. The three recognized chemostratigraphic horizons are: (1) maximum modern Pb pollution; (2) onset of Zn enrichment; and (3) onset of Pb enrichment (Fig. 2).

### Biomarkers

**Fig. 3:**
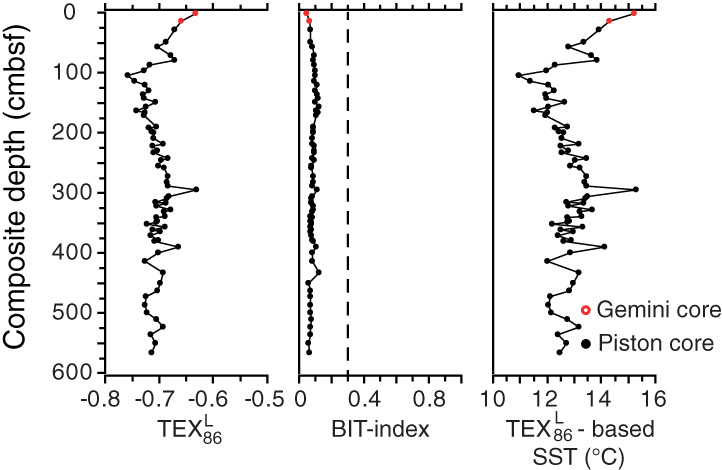
Overview of TEX_86_^L^ values, BIT‐index values and TEX_86_^L^‐based sea surface temperature (SST) reconstructions, using the Baltic Sea calibration by Kabel et al. (2012), for Erstaviken plotted against depth. The dashed line indicates the cut‐off value for the BIT‐index of 0.3 above which TEX_86_^L^‐based SST reconstructions might be obscured by soil organic matter derived GDGTs (Weijers et al. 2006).

All samples contain abundant GDGTs, enabling calculation of TEX_86_^L^ and the BIT‐index (Fig. 3). Values for the BIT‐index fluctuate between 0.05 and 0.12, substantially below the cutoff value for the BIT‐index of 0.3 above which TEX_86_^L^‐based SST reconstructions might be obscured by soil organic matter derived GDGTs (Weijers et al. 2006). Values for TEX_86_^L^ fluctuate between −0.63 and −0.76, corresponding to SSTs between ∼ 11°C and 15°C applying the Kabel et al. (2012) TEX_86_^L^‐calibration for the Baltic Sea. Reconstructed SSTs are around 12.5°C at the base of the record and gradually increase to average values of ∼ 13.5°C, halfway the record, where a maximum in SST is observed of ∼ 15°C. From this point onward, SSTs gradually decrease, reaching a minimum of ∼ 11°C around 100 cmbsf, after which SSTs rapidly increase again to a value of ∼ 15°C at the top of the record.

### Core chronology

**Fig. 4:**
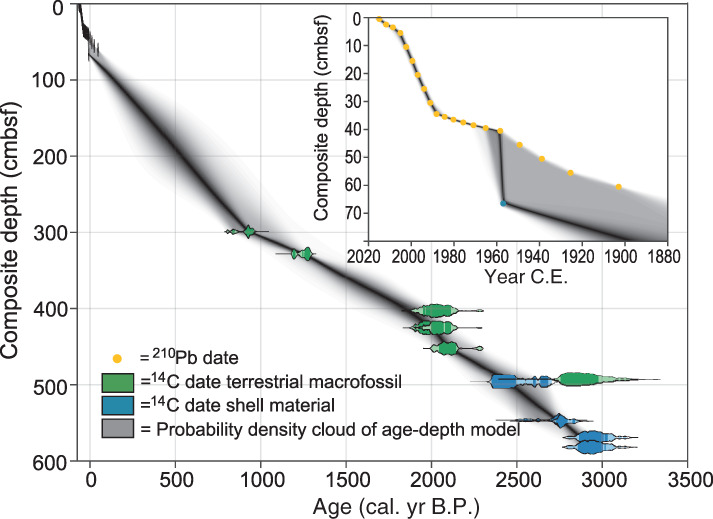
Age‐depth model for Erstaviken. Yellow dots represent ^210^Pb dates (constant rate of supply model; Appleby and Oldfield, 1978) for the top sediments (Gemini core). Silhouettes represent probability distributions of ^14^C dates following age‐depth modeling carried out using the *Undatable* age‐depth modeling software (Lougheed and Obrochta 2019) for terrestrial macrofossils (green) and (fragments of) bivalves (blue), picked from the piston core. The dark shaded area represents a probability density cloud of the *Undatable* age‐depth model, where darker colors indicate a higher probability.

The age‐depth points largely show a typical downcore sequence of progressively increasing age with depth, from 2015 c.e. (the top ^210^Pb date) in the surface sediment to ∼ 3 cal. ka BP at 582 cmbsf (the lowest ^14^C date) and two depths where the sedimentation rate significantly changes, that is, around 35 cmbsf and around 300 cmbsf, respectively (Fig. 4; Supplementary Tables S5 and S6). However, in two instances age‐depth points show some apparent disagreement in the form of age‐depth reversals. The most apparent disagreement is between the youngest *M. balthica* ^14^C date at 66.5 cmbsf (Fig. 4; Supplementary Table S6) and the overlying ^210^Pb dates (Fig. 4; Supplementary Table S5). The ^14^C date is particularly robust, as it is possible to very precisely calibrate its ^14^C activity due to it occurring during the ^14^C production spike associated with atmospheric nuclear weapons testing (i.e., the “bomb peak”). Even if the reservoir age estimation were to be completely wrong for the sample in question (i.e., off by > 200 ^14^C yr), due to the very high atmospheric ^14^C concentration during the bomb peak, the calibrated age of the sample would only shift by one calendar year at most. Furthermore, we can also categorically rule out any possibility of influence of atmospheric contamination during the graphitization and measurement process in the laboratory, because the ^14^C atmospheric activity of the measurement year (2017 c.e.) is significantly lower than that of 1957 c.e., the calibrated age of the sample. One remaining possibility is that the ontogenetic age of the mollusk shell in question is older than its ^14^C age due to secondary carbonate phases. However, the material was pre‐treated, so it should not be affected by such secondary carbonate phases. It is also possible that the calibration of the ^210^Pb age‐depth relationship is incorrect, or that bioturbation affected bulk sediment and mollusk material in different ways. Regardless of the reason for the apparent disagreement, the uncertainty in the age‐depth model, as calculated by *Undatable*, is increased for the interval in question.

Further downcore, at 493.3 and 496.3 cmbsf, a terrestrial macrofossil and mollusk shell exhibit a significant age‐depth reversal. This disagreement can be due to one of a number of reasons: (1) the presence of a ^14^C activity plateau between ∼ 2.7 cal. ka BP and ∼ 2.3 cal. ka BP, which means that precise calibration of ^14^C ages from this interval is difficult; (2) differential bioturbation of mollusk material and the terrestrial macrofossil material; (3) dhe transit time of terrestrial macrofossil material from the catchment to the core site may mean that the terrestrial macrofossil material is older than the core sediment accumulation age; and (4) the reservoir age applied to the mollusk material is too old (however, a test run whereby the reservoir age was halved for the mollusk material did not resolve this problem). In any case, the presence of the age‐depth reversals justifies our application of 20% bootstrapping to incorporate more uncertainty in the *Undatable* age‐depth model.

### Salinity estimates

Salinity estimates, based on ^87^Sr/^86^Sr of the mollusk shells (Supplementary Table S6), are around 10 in the lower part of the record (i.e., between ∼ 600 and 500 cmbsf) and around 6.5 in the upper part of the record (66.5 cmbsf).

## *Discussion*

### Bottom water redox conditions

In well‐oxygenated aquatic environments, Mo is mainly present in dissolved form as the conservatively behaving molybdate ion (MoO_4_^2−^) and is therefore not sequestered in sediments. When sulfide is present in pore or bottom waters, molybdate ions are converted into particle reactive thiomolybdates (MoS_x_O^2−^_x − 4_), enabling sequestration and burial of Mo (Helz et al. 1996; Tribovillard et al. 2006, and references therein). As a result, sedimentary enrichments of Mo are widely applied to reconstruct bottom water redox conditions (Scott and Lyons 2012). Recently, a compilation of sedimentary Mo concentrations in modern sulfidic environments showed that there is a strong bimodal distribution, with Mo concentrations above the crustal average (∼ 1–2 ppm; Turekian and Wedepohl 1961) and below 25 ppm representing settings where dissolved sulfide remained restricted to the pore waters while Mo concentrations above 100 ppm represented a persistently sulfidic water column (Scott and Lyons 2012). Interpretation of intermediate values (25–100 ppm) was shown to be more complicated as a result of variable sedimentation rates, intermittently sulfidic conditions, the effect of pH on Mo speciation and depletion of dissolved Mo from the water column following water mass restriction (Scott and Lyons 2012). Based on the age‐depth model (Fig. 4), we conclude that sedimentation rates were relatively constant at Erstaviken, which is also reflected in the high similarity between the raw concentrations and mass accumulation rates of Mo (Supplementary Fig. S2). However, a recent study (van Helmond et al. 2018), based on crossplots of Mo and C_org_ (cf. Algeo and Lyons 2006), has suggested that there is increased depletion of the water column Mo inventory toward the present in the Baltic Sea, with evidence for continuous water mass restriction during hypoxic periods over the last 8000 yr in the Landsort Deep (site M0063 in Fig. 1a), located proximate to our study area. Crossplots of Mo and C_org_ and Cd/Mo vs. Co*Mn (Supplementary Figs. S3 and S4; Algeo and Lyons 2006; Sweere et al. 2016) for Erstaviken suggest that the water mass in the Stockholm Archipelago was restricted over the last 3000 yr as well; hence, less sedimentary enrichment of trace metals (e.g., Mo) during hypoxic episodes is expected in the Stockholm Archipelago compared with similar hypoxic conditions in an open oceanographic setting. We might therefore underestimate the magnitude of the reducing conditions.

**Fig. 5:**
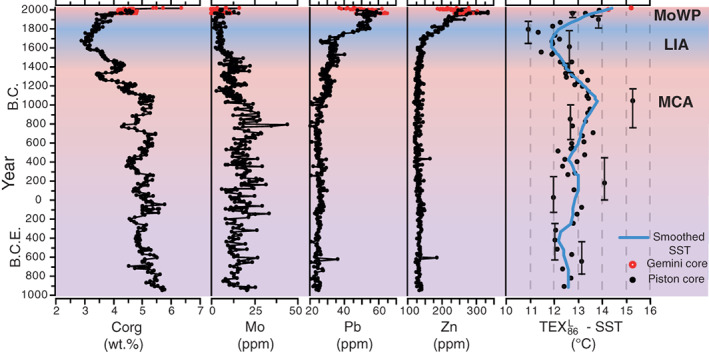
Sedimentary concentrations of organic carbon (C_org_), molybdenum (Mo), lead (Pb), and zinc (Zn) and TEX_86_^L^‐based sea surface temperature (SST) reconstructions, using the Baltic Sea calibration (Kabel et al. 2012), for Erstaviken plotted against the median age of the age‐depth model (Fig. 4). The three climatic intervals discussed, i.e., the Medieval Climate Anomaly (MCA), the Little Ice Age (LIA), and Modern Warm Period (MoWP), are indicated by red, blue, and red shading, respectively. The smoothed SST record was generated by a locally weighted regression fitting using a 2^nd^ order polynomial, with a span set to 40% of the temporal length of the record. Error bars on the 10 selected data points indicate minimum and maximum ages of the age‐depth model.

At Baggensfjärden and Ingaröfjärden, the initially low Mo concentrations are indicative of initially well‐oxygenated, non‐sulfidic conditions (Fig. 2b,c). The relatively high sedimentary concentrations (at the base of the record at Erstaviken, and halfway through the records of Baggensfjärden and Ingaröfjärden; Fig. 2; Supplementary Fig. S2) represent a period of pronounced oxygen depletion in the Stockholm Archipelago with sulfide continuously being present in the pore waters and potentially (occasionally) also in the bottom waters, that is, euxinic conditions. For Erstaviken, this period of pronounced oxygen depletion lasted from at least 1000 b.c.e. until ∼ 1500 c.e. (Fig. 5). At all three sites, the relatively high C_org_ content indicates a high input of organic matter for the entire study interval, with the exception of the presumably post‐glacial lacustrine clay layer in the lower part of Baggensfjärden. The low BIT‐index values (< 0.3) for Erstaviken (Fig. 3) show that most of the organic matter is of marine origin, suggesting high local marine primary productivity (Hopmans et al. 2004).

**Fig. 6:**
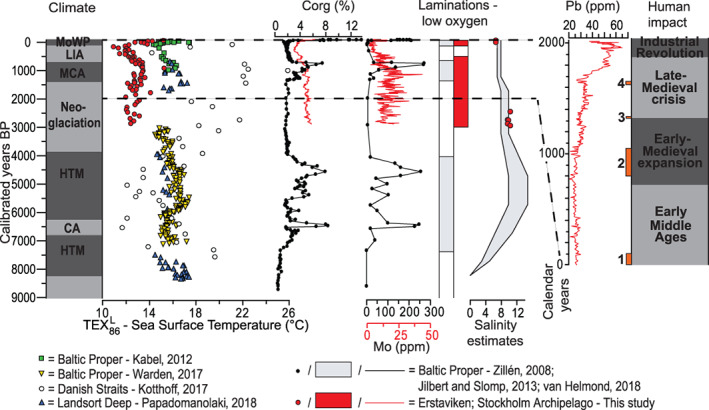
Comparison of the records generated for Erstaviken (this study) with a selection of published records for the Holocene (~ last 9000 yr) Baltic Sea, with from left to right: climate zones (cf. Zillén and Conley 2010; HTM = Holocene thermal maximum, CA = climate anomaly, MCA = Medieval Climate Anomaly, LIA = Little Ice Age, MoWP = Modern Warm Period), TEX_86_^L^‐based sea surface temperatures from the Baltic proper (Kabel et al. 2012; Warden et al. 2017), Danish straits (Little Belt – M0059; Kotthoff et al. 2017) and Landsort Deep (M0063; Papadomanolaki et al. 2018), sedimentary organic carbon (C_org_), molybdenum (Mo), and laminations/low oxygen for the Baltic proper (Jilbert and Slomp 2013; van Helmond et al. 2018), salinity estimates for the Baltic proper (Zillén et al. 2008) and human impact over the last ~ 2000 yr (modified after Zillén and Conley 2010), including events discussed: (1) Greek‐Roman period; (2) Viking age; (3) black death; and (4) Thirty Years War.

When Mo concentrations in the study area are compared with Mo concentrations in other coastal areas in the Baltic Sea during the Holocene, it becomes clear that absolute Mo concentrations in the Stockholm Archipelago prior to modern hypoxia are generally somewhat higher (avg. 20, max. 44 ppm for Erstaviken; avg. 25, max. 43 for Baggensfjärden; and avg. 10, max. 14 for Ingaröfjärden) than in the Danish Little Belt over the past 8000 yr (avg. 7 ppm, max. 20 ppm; van Helmond et al. 2017) and the Finnish Archipelago Sea over the past 1500 yr (< 10 ppm; Jokinen et al. 2018). Molybdenum concentrations generally remain below 25 ppm, however, suggesting that dissolved sulfide was present but remained restricted to the pore waters most of the time in all the studied coastal areas (Scott and Lyons 2012). During the HTM and MCA in the Baltic Proper, absolute Mo concentrations are up to an order of magnitude higher when compared to the coastal areas, for example, Erstaviken (Fig. 6). These high values are indicative of persistent euxinia in the Baltic Proper and clearly illustrate the large contrast in bottom water redox conditions between the open Baltic Sea and its coastal zone, which can be attributed to the strong halocline in the Baltic Proper that obstructs deep water mixing (Jilbert and Slomp 2013; Papadomanolaki et al. 2018; van Helmond et al. 2018). The high variability in the Mo record for Erstaviken shows that bottom water oxygen concentrations were highly dynamic. The higher and more stable Mo values for Baggensfjärden suggest that this site was more often euxinic, whereas lower Mo values for Ingaröfjärden show that sulfide always remained restricted to the pore waters at this location (Scott and Lyons 2012). This spatial trend in the reconstructed bottom water redox conditions strongly resembles that of the present‐day conditions at our study sites (Table 1). We attribute this bottom water redox gradient to the different water depths and geography at the three sites, with the highest degree of restriction of water flows in Baggensfjärden (Fig. 1b). We can exclude a role for the distance to the city of Stockholm as a cause for the redox gradient, because of restricted hydrological connections between our study area and the inner Archipelago. Furthermore, Ingaröfjärden provides the primary hydrological connection to Baggensfjärden and Erstaviken (Almroth‐Rosell et al. 2016).

From ∼ a.d. 1500 onward, Mo decreases at Erstaviken (Figs. 5, 6), presumably simultaneous with the decrease in Mo at Ingaröfjärden and Baggensfjärden (Fig. 2). Molybdenum levels decrease to values around the crustal average, indicative of well‐oxygenated conditions. The timing of the improvement in bottom water redox conditions in the Stockholm Archipelago corresponds to the termination of widespread oxygen depletion in the Baltic Proper (Jilbert and Slomp 2013; Fig. 6). This suggests a common driver for the coastal zone and the open Baltic Sea, for example, climate and/or nutrient recycling (Kabel et al. 2012; Jilbert and Slomp 2013; Funkey et al. 2014; Papadomanolaki et al. 2018). From ∼ 1750 c.e. onward, C_org_ increases again at Erstaviken (Figs. 5, 6), presumably simultaneous with a similar increase in C_org_ at Ingaröfjärden, whereas Mo remains low at all three sites (Fig. 2), indicating that while organic matter input increased again, bottom waters remained well‐oxygenated. Enrichments in Mo, reflecting a return of low oxygen bottom waters, only occurs again in the top sediments at Bäggensfjärden and Erstaviken, that is, over the course of the last century. This is in accordance with previous findings (Jonsson 2003; Conley et al. 2011).

### Human activity

Despite much earlier evidence for cereal cultivation, the Stockholm Archipelago region only became a prominent area for large‐scale international trade in the Viking Age (∼ 800–1050 c.e.), a period of expanding agriculture and stock‐farming in which the first town of Sweden, Birka (∼ 750 c.e.; ∼30 km west of Stockholm), became a permanent settlement (Risberg et al. 2002; Myrdal and Morell 2011; Kalmring 2016). The large European population boom and expansion from 1000 to 1300 c.e. is linked to more than a doubling in cropland area in Europe from 800 to 1300 c.e. (McEvedy and Jones 1978; Pongratz et al. 2008). Tentative population estimates for the Baltic Sea area for this period suggest a doubling in population from 4.6 to 9.5 million, inferred to correspond with increases in nutrient loading (Renberg et al. 2001; Zillén and Conley 2010). The European population steeply declined, by at least one‐third, as a result of the Black Death, which resulted in the large‐scale abandonment of farms in Sweden (Antonson 2009; Myrdal and Morell 2011). The European population did not recover until ∼ 1500 c.e., after which the population gradually increased from 1500 c.e. till 1700 c.e., followed by another phase of rapid expansion, with a six fold increase in the population of Europe and the Baltic Sea region from 1700 c.e. to recent times (McEvedy and Jones 1978). In particular, the phase since 1700 c.e. is associated with an increase in arable land in Sweden (Myrdal and Morell 2011).

Sedimentary Pb is widely applied as a chronological marker and indicator of human activity in peats, soils, and sediments. Lead‐pollution can be very local, but also regional, depending on the source of the Pb‐pollution, which can be waterborne (relatively local) or result from atmospheric transport, that is, a more regional source (Renberg et al. 1994; Bindler et al. 1999; Bindler et al. 2009). The background concentrations of Pb (Pb > 20 ppm) in the three studied records in the Stockholm Archipelago (Fig. 2) are relatively high, which might explain the absence of the pollution peak characteristic for the Greek‐Roman period (∼ 0–100 c.e.), observed in most lake and peat records in Sweden (Brännvall et al. 1999; Renberg et al. 2000; Bindler et al. 2009). The timing of the first increase in Pb concentrations (“3” in Fig. 2) in the Stockholm Archipelago (∼ 1000 c.e. for Erstaviken; Fig. 5) corresponds with the onset of pronounced Pb‐pollution in lake sediments throughout Sweden during the Viking Age (Brännvall et al. 1999; Bindler et al. 2009; Fig. 6). ^206^Pb/^207^Pb isotope ratios from a suite of records from central Sweden show that the Pb‐pollution is a mixture of both waterborne and atmospheric sources, partly originating from large‐scale mining in the Bergslagen area in central Sweden (Renberg et al. 2002; Bindler et al. 2009). Archeological and historical evidence also indicate increased human activity in the Stockholm region, which had become a prominent area for large‐scale international trade and politics, around that time (Risberg et al. 2002; Kalmring 2016). The remainder of the sedimentary Pb record from the Stockholm Archipelago largely follows the trends observed in Swedish lakes and peats (Brännvall et al. 1999; Renberg et al. 2000; Renberg et al. 2002). This includes the depression in Pb related to the Black Death (∼ 1350 c.e.; Fig. 6), a small minimum potentially linked to the Thirty Years War (1618–1648 c.e.; Fig. 6) and the subsequent large and rapid increase in the 18^th^ century (Brännvall et al. 2001) that is linked to the start of the modern population boom just preceding the Industrial Revolution (McEvedy and Jones 1978). The peak in Pb concentrations observed toward the top of the records in the Stockholm Archipelago (∼ 1970 c.e.) is related to atmospheric Pb‐deposition following modern industrialization and the use of leaded petrol (Brännvall et al. 1999; Renberg et al. 2000; Fig. 6).

Besides Pb, the (semi‐)modern use of a range of trace metals in agriculture and industry, for example, in fertilizers, pesticides, pigments, and lubricants, has led to their enrichments in different sedimentary regimes (Zwolsman et al. 1993; Audry et al. 2004). A recent study on trace metals in sediments from the Baltic Proper showed that, besides Pb, particularly Zn is strongly enriched in modern sediments due to anthropogenic activity (van Helmond et al. 2018). In the Stockholm Archipelago, sedimentary Zn concentrations remain at background levels until the onset of the 18^th^ century (Fig. 5), after which their increase coincides with that of Pb, possibly linked to the onset of the modern population boom (McEvedy and Jones 1978). A similar increase in Zn was observed in sediments from Gåsfjärden, about 300 km south of the Stockholm Archipelago (Ning et al. 2018), but this increase was dated to the 20^th^ century and attributed to Zn release from vehicle tire wear (Davis et al. 2001). This suggests that the Stockholm Archipelago Zn increase must have had a different origin, which might well have been the mines in the Bergslagen area in central Sweden, upstream of the Stockholm Archipelago (Bindler et al. 2012).

### Climate

Climatic warming may contribute to the development of hypoxia by (1) decreasing oxygen gas solubility (2) enhancing respiration rates and (3) promoting water column stratification, inhibiting mixing of the water column (Keeling and Garcia 2002; Paerl and Huisman 2009). In the Baltic Sea, enhanced primary productivity during previous warm periods is attributed to increased rates of phosphorus recycling from Baltic Sea sediments due to the hypoxia (Conley et al. 2002; Funkey et al. 2014).

Absolute TEX_86_^L^‐based SST reconstructions for Erstaviken over the past 3000 yr (Fig. 5) are somewhat lower than previous TEX_86_^L^‐based SST reconstructions for the Baltic Sea during the same period (Kabel et al. 2012; Warden et al. 2017, Kotthoff et al. 2017; Papadomanolaki et al. 2018; Fig. 6). The lower SST estimates for the Stockholm Archipelago compared to those for the Baltic Proper are in line with the modern, measured gradient in SSTs for the Baltic Sea, where SSTs are 2°C to 3°C higher in the Baltic Proper than in the Stockholm Archipelago (Siegel et al. 2006).

Trends in the reconstructed SST record for Erstaviken over the past 3000 yr (Figs. 5, 6), including warming toward the MCA, cooling toward the Little Ice Age (LIA) and modern warming (Modern Warm Period), closely resemble those of previous SST reconstructions for the Baltic Sea (Kabel et al. 2012; Kotthoff et al. 2017; Papadomanolaki et al. 2018; Fig. 6) as well as trends in Northern Hemispheric and global temperature records (Moberg et al. 2005; Mann et al. 2009).

The absolute changes in temperature in the Stockholm Archipelago match those in the Baltic Proper and Landsort Deep (Kabel et al. 2012; Papadomanolaki et al. 2018), indicating that the SST‐evolution in the Stockholm Archipelago went hand in hand with that in the more open, deeper parts of the Baltic Sea.

### Drivers of hypoxia in the coastal Baltic Sea

The sedimentary records of Mo (Figs. 2, 5, 6; Supplementary Fig. S2) indicate that the Stockholm Archipelago was (seasonally) hypoxic and potentially occasionally euxinic for a period of at least two and a half millennium (1000 b.c.e. to 1500 c.e.) over the past 3000 yr (based on the age‐depth model for Erstaviken). This premodern hypoxic phase does not correspond with marked changes in human activity or climate in this area, suggesting a different (natural) cause. Such a cause could be high natural nutrient loading (van Helmond et al. 2017), or may be related to the higher bottom water salinity at that time (Fig. 6; Supplementary Table S6), which would have led to stronger water column stratification and consequently reduced bottom water ventilation.

Existing archeological and paleobotanical records show a large increase in human activity in the region from the start of the Viking Age (∼ 800–1050 c.e.) onward (McEvedy and Jones 1978; Myrdal and Morell 2011), which is also reflected by increasing Pb concentrations in the sediments of the Stockholm Archipelago (Figs. 2, 5, 6). Despite increasing human activity, no clear change in sedimentary Mo is observed (Figs. 5, 6; Supplementary Fig. S2). From 1500 c.e. onward, sedimentary Mo clearly decreases, indicative of improved bottom water redox conditions, which coincides with cooler SSTs during the LIA (Figs. 5, 6). These records suggest that Medieval human activity did not lead to (further) deterioration of the water quality in the Stockholm Archipelago, but that climatic cooling, and perhaps lower bottom water salinity (leading to weakened stratification, hence enhanced ventilation; Fig. 6; Supplementary Table S6; Ning et al. 2016), led to the observed recovery from hypoxia at this time. This points toward an important role for climate as a contributor to hypoxic conditions in the coastal Baltic Sea. Indeed, the lowest concentrations of Mo and C_org_ occur simultaneously with the lowest reconstructed SSTs (Figs. 5, 6). The onset of the subsequent rapid rise in SSTs coincides with increased sedimentary concentrations of C_org_, Pb and Zn related to higher primary productivity, the modern rise in population (from 1700 c.e. onward) and the Industrial Revolution (McEvedy and Jones 1978; Zillén et al. 2008). Hypoxic conditions nevertheless do not return prior to the 20^th^ century, the modern, human‐induced hypoxic phase (Gustafsson et al. 2002; Carstensen et al. 2014).

## *Conclusion and implications*

Our results underline that the timing of past coastal hypoxia in the Baltic Sea differs from that in offshore areas, and that changes in climate and (potentially) salinity were instrumental in its cause. Recent model sensitivity studies have shown that anthropogenic nutrient loading was key for the development of modern hypoxia in offshore areas of the Baltic Sea (Meier et al. 2018). Nutrient reduction therefore provides a key pathway to recovery, as shown recently for part of the Stockholm Archipelago as well (Karlsson et al. 2010). Because temperature is such an important factor in determining the presence or absence of hypoxia in the Stockholm Archipelago over the past ∼ 3000 yr, ongoing global warming has the potential to counterbalance the positive effects of current nutrient reductions. This may impede recovery of the Stockholm Archipelago to well‐oxygenated conditions. In order to account for the negative effects of ongoing global warming nutrient reduction schemes might need to become more ambitious. The exact role of long‐term changes in salinity remains unresolved. But in general, freshening of the coastal Baltic Sea positively influences bottom water redox conditions, both by weakening stratification and increasing the potential for sediment nutrient sequestration (Lenstra et al. 2018).

## Conflict of Interest

None declared.

## Supporting information


**Appendix S1**: Supporting Information
