# Supplementary material for: Recovery from multi‐millennial natural coastal hypoxia in the Stockholm Archipelago, Baltic Sea, terminated by modern human activity
Source: Limnol Oceanogr. 2020 Aug 18;65(12):3085–97. doi: 10.1002/lno.11575 (PMC7754161; doi:10.1002/lno.11575)
Supplement: Supplementary file 1 — Appendix S1: Supporting Information [file LNO-65-3085-s001.docx]

**Supplementary information**

**Recovery from multi-millennial natural coastal hypoxia in the Stockholm Archipelago, Baltic Sea, terminated by modern human activity**

Niels A. G. M. van Helmond^1, 2, *^, Bryan C. Lougheed^3,4^, Annika Vollebregt^1^, Francien Peterse^1^, Guillaume Fontorbe^2,a^, Daniel J. Conley^2^, and Caroline P. Slomp^1^

^1^ Department of Earth Sciences, Faculty of Geosciences, Utrecht University. Princetonlaan 8a, 3584 CB Utrecht, The Netherlands. ^2^ Department of Geology, Lund University, Sölvegatan 12, 22362 Lund, Sweden. ^3^ Department of Earth Sciences, Uppsala University, Villavägen 16, 75236 Uppsala, Sweden. ^4^ Laboratoire des Sciences du Climat et de l'Environnement, LSCE/IPSL, CEA CNRS-UVSQ, Université Paris-Saclay, 91190 Gif-sur-Yvette, France. ^a^ present address: GEOMAR, Helmholtz-Zentrum für Ozeanforschung Kiel, Wischhofstrasse 1-3, 24148 Kiel,Germany. ^*^ Corresponding author: n.vanhelmond@uu.nl

**Sediment analysis**

**Organic carbon content**

Per sample (n=119 for Ingaröfjärden, n=430 for Erstaviken, n=119 for Baggensfjärden; Table S1-3) approximately 0.3 g of freeze-dried and powdered sediment was weighed into 15 ml centrifuge tubes. Subsequently, the sediments were decalcified using HCl. After addition of 7.5 ml of 1M HCl, samples were put on a shaker. After 4 hours the HCl was removed upon centrifugation and another 7.5 ml of 1M HCl was added before placing the samples back on the shaker. The following day the acid was removed again upon centrifugation after which the samples were washed twice with milliQ water, and dried in an oven programmed at 60°C for 72 hours. Dry residues were then reweighed to determine the weight loss due to decalcification. Before analysis the samples were powdered and homogenized again and between 10 and 20 mg per sample was weighed into tinfoil cups. Total carbon analyses were performed using a Fisons Instruments NA 1500 NCS analyzer at Utrecht University. Obtained results were normalized to in-house standards, acetanilide, atropine and nicotinamide. Accuracy and precision of analyses were determined based on measurements of the internationally certified soil standard IVA2 after every 10 to 20 samples. The certified value for IVA2 is 0.732 wt.% C, the mean value that we obtained for IVA2 (n=41) was 0.714 wt.% C with a standard deviation of 0.024 wt.% C. Afterwards Corg was calculated after correction for the weight loss due to decalcification and the salt content of the freeze dried sediment samples. An average analytical uncertainty of 0.06 wt.% was calculated based on duplicate analyses of sediment samples (n=42).

**Total elemental composition**

To determine sedimentary elemental concentrations, per sample (n=119 for Ingaröfjärden, n=430 for Erstaviken, n=119 for Baggensfjärden; Table S1-S3) approximately 125 mg of freeze-dried and powdered sediment was weighed in 30 ml Teflon vessels after which 2.5 ml of mixed acid (HClO_4_:HNO_3_, 3:2) and 2.5 ml 40% HF were added before the vessels were left overnight on a hotplate at 90°C. The following day the lids of the vessels were removed and the extracts were heated to 140°C to evaporate the acids. The remaining residues were then dissolved in 25 ml 4.5% HNO_3_ and left overnight on a hotplate at 90°C. The dilution of the final solutions was determined by weighing the vessels, after which the solutions were analyzed by Inductively Coupled Plasma-Optical Emission Spectrometry (ICP-OES; SPECTRO ARCOS) at Utrecht University. The accuracy (recovery) was between 95 and 105% for molybdenum (Mo) and zinc (Zn), between 96 and 116% for lead (Pb), between 96 and 103% for aluminum (Al), between 97 and 104% for sulfur (S), between 98 and 103 for cadmium (Cd), between 99 and 105 for cobalt (Co) and between 96 and 101 for manganese (Mn) based on in-house standards. The average analytical uncertainty based on duplicate analyses of sediment samples (n=103) was 1 part per million (ppm) for Mo, 3 ppm for Pb, 5 ppm for Zn, 1617 ppm for Al, 569 ppm for S, <1 ppm for Cd and Co and 305 ppm for Mn.

**Biomarkers**

For Erstaviken approximately 1 g of freeze-dried and powdered sediment (n=69; Figure S5; Table S1) was extracted with a dichloromethane (DCM): Methanol (MeOH) (9:1; v/v) solvent mixture, using an Accelerated Solvent Extractor (ASE350, DIONEX) at a temperature of 100°C and a pressure of 7.6 × 10^6^ Pa. Total lipid extracts were separated into an apolar, ketone and polar fraction using Al_2_O_3_ column chromatograhphy using hexane:DCM (9:1, v/v) hexane:DCM (1:1, v/v) and MeOH:DCM (1:1, v/v), respectively. To each of the polar fractions, 99 ng of the C_46_ glycerol dialkyl glycerol tetraether (GDGT) internal standard was added, dried under a N_2_ flow, after which they were redissolved in hexane:isopropanol (99:1, v/v) to a concentration of ~2 mg ml^−1^ and filtered over a 0.45 μm PTFE filter. The filtered fraction was then analyzed by ultra-high-performance liquid chromatography-mass spectrometry (UHPLC-MS) on an Agilent 1260 infinity series instrument coupled to a 6130 quadrupole mass selective detector at Utrecht University with settings as described in Hopmans et al. (2016) to determine the abundances of the different GDGTs.

In order to translate GDGT abundances to sea surface temperatures (SSTs) TEX_86_^L^ values were determined according to Kim et al. (2010) after which SSTs were calculated using the calibration for the Baltic Sea by Kabel et al. (2012). The latter calibration showed that the best correlation was obtained for average SSTs from July to October. The average analytical uncertainty, based on duplicate measurements of polar fractions (n=3), was 0.01 for TEX_86_^L^, corresponding to 0.37°C in SST.

To determine whether SST reconstructions based on TEX_86_^L^ for the Stockholm Archipelago may have been biased by the input of soil organic matter derived GDGTs (Weijers et al. 2006) the Branched and Isoprenoid Tetraether (BIT) index according to Hopmans et al. (2004) was calculated.

**Core chronology**

**Lead-210 (^210^Pb) dating**

Freeze-dried sediment was measured for ^210^Pb, ^226^Ra and ^137^Cs by direct gamma counting using a high purity germanium detector (Ortec GEM-FX8530P4-RB) at Lund University. ^210^Pb was measured by its emission at 46.5 keV, ^226^Ra by the 351 keV emission by its daughter isotope ^214^Pb, ^137^Cs was measured by its emissions at 661 keV. Self-absorption was measured directly and the detector efficiency was determined by counting a National Institute of Standards and Technology (NIST) sediment standard. Excess ^210^Pb was calculated as the difference between the measured total ^210^Pb and the estimate of the supported ^210^Pb activity as given by ^214^Pb (^210^Pb_exc_ = ^210^Pb_total_ − ^214^Pb). The age–depth relationship was calculated using both the constant initial concentration (CIC) model and the constant rate of supply (CRS) model (Appleby and Oldfield 1978) of which the latter assumes that sedimentation rate and sediment compaction change throughout the core and automatically corrects for these parameters. ^210^Pb-dates for both models are provided in Table S4. For the age model of Erstaviken, CRS dates based on the best fit to the ^137^Cs profile were used. The dating of the sediment layers was constrained by the agreement between the ^210^Pb chronology and the 1986 CE Chernobyl reactor explosion.

**Strontium isotope determinations**

For Erstaviken ^87^Sr/^86^Sr ratios (Table S5) on carbonate material were determined at the facilities of the Swedish Museum of Natural History (Naturhistoriska riksmuseet - NRM) in Stockholm. Terrestrial and marine macrofossils were first chemically leached with weak acetic acid (HAc; 0.1 M) to remove any secondary carbonate phases, and then rinsed multiple times in purified water. Subsequently, 1-2 mg of pre-treated carbonate was dissolved in 2.5 ml 1M HAc at 70°C, centrifuged, dried down and treated with concentrated HNO_3_. The dried-down sample was dissolved in 2 ml 6M HCl and Sr was subsequently separated using NRM’s “WRS-1” (Sr separation) and “WRS-2” (Sr separation from Ba) standard ion exchange column chemistry procedures, using a mix of Triskem SR-B100S and TR-B200S resins, after which it was dried down and treated with H_2_O_2_. Thereafter, 100 – 200 ng of extracted Sr material was loaded onto Re filaments, using a TaCl_2_ activator to improve ionisation, after which the loaded filaments were analyzed for ^87^Sr/^86^Sr ratios using a Thermo TRITON Plus thermal ionisation mass spectrometer (TIMS). All reported ^87^Sr/^86^Sr ratios have been Rb-corrected (^87^Rb/^85^Rb = 0.38600) and normalised to the NBS987 value of 0.710245, based on standard measurements of 0.710220 ± 15 (2σ, n=12). Procedural blanks resulted in 21 pg or less of Sr, which has a negligible effect upon the isotopic ratio analysis.

**Radiocarbon (^14^C) dating**

To avoid the pitfalls associated with ^14^C dating of bulk sediment in the Baltic Sea (Olsson, 1979; Lougheed et al. 2017), we concentrated exclusively on macrofossils (terrestrial samples and mollusc shells). ^14^C determinations (Table S5) for all samples were carried out using a ^14^C single stage accelerated mass spectrometer (SSAMS) at Lund University. Radiocarbon reservoir age, or R(t), for mollusc macrofossils in the hydrographically dynamic Baltic Sea can vary greatly in both time and space (Lougheed et al. 2013). Aware of this issue, we used our ^87^Sr/^86^Sr measurements on *Macoma balthica* to apply a previously developed ^87^Sr/^86^Sr-based R(t) transfer function (Lougheed et al. 2016), thus resulting in a unique R(t) estimate for each *M. balthica* macrofossil. All but one of the ^14^C dates, both marine and terrestrial, were calibrated to the atmospheric IntCal13 (Reimer et al. 2013) calibration curve using the MatCal calibration software (Lougheed and Obrochta 2016). This includes mollusc shells, which are highly influenced by atmospherically equilibrated river runoff in the Baltic Sea. A single post-bomb ^14^C determination (LuS12149) was calibrated by looking up its reservoir age corrected ^14^C activity in both the pre-bomb peak and post-bomb peak sections of the Bomb13NH1 (Hua et al. 2013) calibration curve. The two possible calibrated ages for this ^14^C determination are 1957 CE and 2003 CE. Based on the position of the sample in the sediment sequence, 2003 CE was subsequently excluded as a possibility.

.

**Supplementary figures**


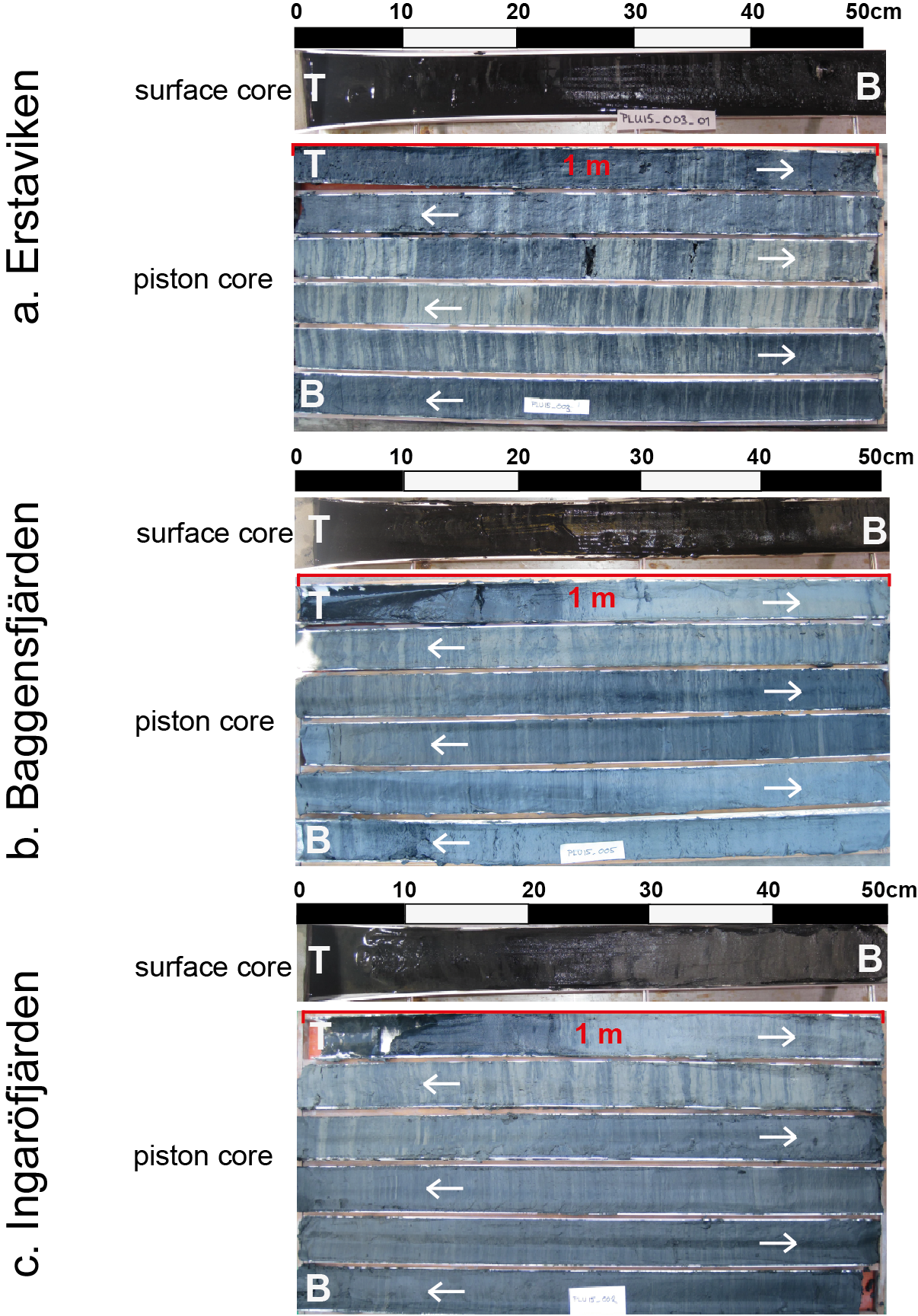


**Figure S1.** Core photographs of the three studied sites.


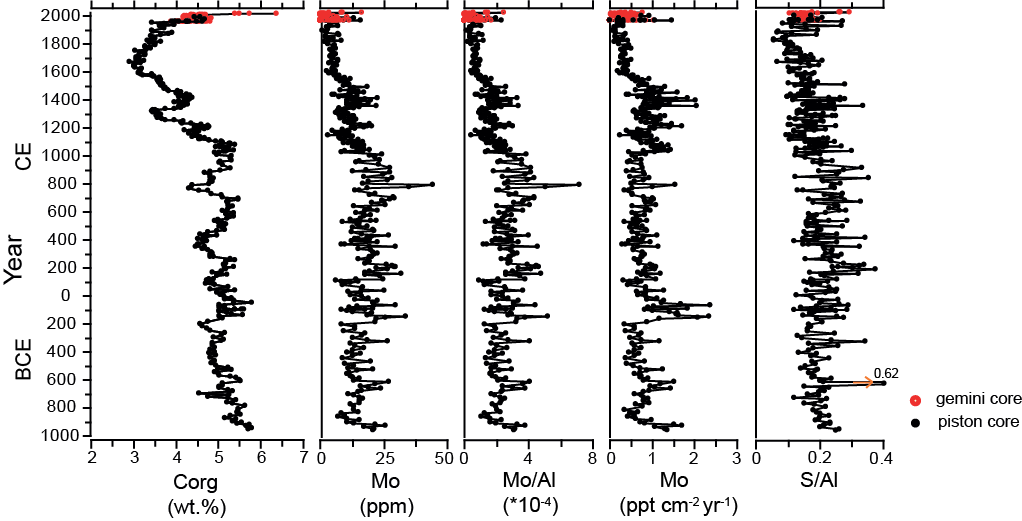


**Figure S2.** Sedimentary concentrations of organic carbon (Corg), molybdenum (Mo), Mo/Al, mass accumulation rates (MARs) of Mo and S/Al for Erstaviken plotted against the median age of the age-depth model presented in this study.


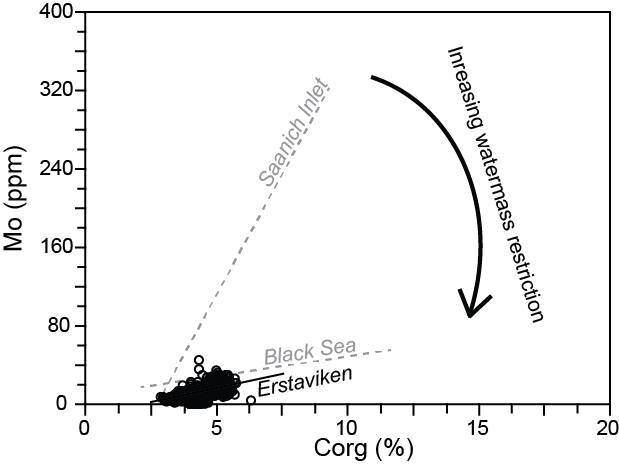


**Figure S3.** Crossplot of sedimentary Mo versus Corg for Erstaviken. The dashed lines show the regression slopes for sedimentary Mo versus Corg for the weakly restricted Saanich Inlet and the strongly restricted Black Sea (Algeo and Lyons, 2006).


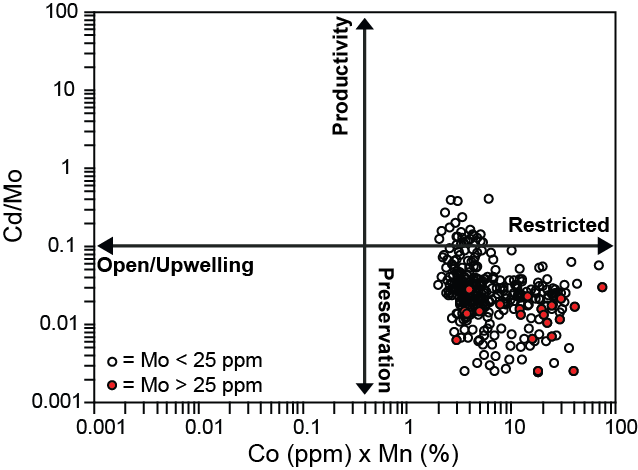


**Figure S4.** Crossplot of sedimentary Cd/Mo versus Co*Mn for Erstaviken. The arrows give an indication of the depositional settings in which the corresponding values can be found (see Sweere et al., 2016 for details). Samples in red contain Mo > 25 ppm, which is thought to be indicative of at least occasionally euxinic conditions (Scott and Lyons, 2012).

**References**

Algeo, T. J., and T. W. Lyons. 2006. Mo–total organic carbon covariation in modern anoxic marine environments: Implications for analysis of paleoredox and paleohydrographic conditions. Paleoceanography 21(1), PA1016, doi:10.1029/2004PA001112.

Appleby, P. G., and F. Oldfield. 1978. The calculation of lead-210 dates assuming a constant rate of supply of unsupported ^210^Pb to the sediment. Catena 5, 1–8, doi:10.1016/S03418162(78)80002-2.

Hopmans, E. C., J. W. H. Weijers, E. Schefuß, L. Herfort, J. S. Sinninghe Damsté, and S. Schouten. 2004. A novel proxy for terrestrial organic matter in sediments based on branched and isoprenoid tetraether lipids. Earth Planet. Sci. Lett. 224(1), 107–116, doi:10.1016/j.epsl.2004.05.012.

Hopmans, E. C., S. Schouten, and J. S. Sinninghe Damsté. 2016. The effect of improved chrmatography on GDGT-based palaeoproxies. Org. Geochem. 93, 1–6, doi:10.1016/j.orggeochem.2015.12.006.

Hua, Q.; M. Barbetti, and A. Z. Rakowski. 2013. Atmospheric Radiocarbon for the Period 1950–2010. Radiocarbon 55, 2059–2072, doi:10.2458/azu_js_rc.v55i2.16177.

Kabel, K., M. Moros, C. Porsche, T. Neumann, F. Adolphi, T. J. Andersen, H. Siegel, M. Gerth, T. Leipe, E. Jansen, and J. S. Sinninghe Damsté. 2012. Impact of climate change on the Baltic Sea ecosystem over the past 1.000 years. Nat. Clim. Change 2012 (2), 871–874, doi: 10.1038/nclimate1595.

Kim, J. H., J. van der Meer, S. Schouten, P. Helmke, V. Willmott, F. Sangiorgi, N. Koc, E. C. Hopmans, and J. S. Sinninghe Damsté. 2010. New indices and calibrations derived from the distribution of crenarchaeal isoprenoid tetraether lipids: Implications for past sea surface temperature reconstructions. Geochim. Cosmochim. Acta 74, 4639-4654, doi:10.1016/j.gca.2010.05.027.

Lougheed, B. C., H. L. Filipsson, and I. Snowball. 2013. Large spatial variations in coastal 14C reservoir age–a case study from the Baltic Sea. Clim. Past 9, 1015–1028, doi:10.5194/cp-9-1015-2013.

Lougheed, B. C. and S. P. Obrochta. 2016. MatCal: Open Source Bayesian 14C Age Calibration in Matlab. J. Open Res. Software 4, doi:10.5334/jors.130.

Lougheed, B. C., H. J. L. van der Lubbe, and G. R. Davies. 2016. ^87^Sr/^86^Sr as a quantitative geochemical proxy for ^14^C reservoir age in dynamic, brackish waters: Assessing applicability and quantifying uncertainties. Geophys. Res. Lett. 43, 735–742, doi:10.1002/2015GL066983.

Lougheed, B. C., S. P. Obrochta, C. Lenz, A., Mellström, B. Metcalfe, R. Muscheler, M. Reinholdsson, I. Snowball, and L. Zillén. 2017. Bulk sediment ^14^C dating in an estuarine environment: How accurate can it be?: Estuarine Bulk Sediment ^14^C Dating. Paleoceanography 32, 123–131, doi:10.1002/2016PA002960.

Lougheed, B. C., and S. P. Obrochta. 2019. A rapid, deterministic age-depth modelling routine for geological sequences with inherent depth uncertainty. Paleoceanography and Paleoclimatology 34(1), 122-133, doi:10.1029/2018PA003457.

Olsson, I. 1978. A warning against radiocarbon dating of samples containing little carbon. Boreas 8, 203–207, doi:10.1111/j.1502-3885.1979.tb00801.x.

Reimer, P. J., E. Bard, A. Bayliss, J. W. Beck, P. G. Blackwell, C. Bronk Ramsey, C. E. Buck, H. Cheng, R. L. Edwards, M. Friedrich, et al. 2013. IntCal13 and Marine13 radiocarbon age calibration curves 0–50,000 years cal BP. Radiocarbon 55, 1869–1887, doi:10.2458/azu_js_rc.55.16947.

Scott, C., and T. W. Lyons. 2012. Contrasting molybdenum cycling and isotopic properties in euxinic versus non-euxinic sediments and sedimentary rocks: refining the paleoproxies. Chem. Geol. 324–325, 19–27, doi:10.1016/j.chemgeo.2012.05.012.

Sweere, T., S. van den Boorn, A. J. Dickson, and G. J. Reichart. 2016. Definition of new trace-metal proxies for the controls on organic matter enrichment in marine sediments based on Mn, Co, Mo and Cd concentrations. Chem. Geol. 441, 235-245, doi:10.1016/j.chemgeo.2016.08.028.

Weijers, J. W. H., S. Schouten, O. C. Spaargaren, and J. S. Sinninghe Damsté. 2006. Occurrence and distribution of tetraether membrane lipids in soils: implications for the use of the TEX_86_ proxy and the BIT index. Org. Geochem. 37(12), 1680–1693, doi:10.1016/j.orggeochem.2006.07.018.

**Tables**

**Table S1.** Median calendar age, sedimentary concentrations of organic carbon (Corg), molybdenum (Mo), lead (Pb), zinc (Zn), aluminum (Al), sulfur (S), cadmium (Cd)/Mo, cobalt (Co; ppm)/Manganese (Mn; wt.%) and mass accumulation rates of Mo for Erstaviken.

| **Core** | **Labcode** | **Composite depth (cmbsf)** | **Median calendar age** | **Corg (wt.%)** | **Mo (ppm)** | **Pb (ppm)** | **Zn (ppm)** | **Al (ppm)** | **S (ppm)** | **Cd/Mo** | **Co (ppm)/**  **Mn (wt.%)** | **Mo**  **(ppt**  **cm^-2^**  **yr^-1^)** |
| --- | --- | --- | --- | --- | --- | --- | --- | --- | --- | --- | --- | --- |
| Gemini | MC1 | 0,5 | 2015 | 6,35 | 4 | 42 | 216 | 63031 | 11092 | 0 | 7 | 0,3 |
| Gemini | MC2 | 1,5 | 2013 | 5,74 | 9 | 46 | 243 | 67829 | 18002 | 0 | 5 | 0,4 |
| Gemini | MC3 | 2,5 | 2012 | 5,39 | 9 | 37 | 217 | 58890 | 15441 | 0 | 4 | 0,8 |
| Gemini | MC4 | 3,5 | 2009 | 5,49 | 16 | 46 | 280 | 67577 | 19879 | 0 | 27 | 0,5 |
| Gemini | MC5 | 4,5 | 2007 | 4,63 | 4 | 39 | 187 | 61374 | 12017 | 0 | 3 | 0,2 |
| Gemini | MC6 | 5,5 | 2006 | 4,35 | 1 | 49 | 216 | 77028 | 8152 | 0 | 2 | 0,1 |
| Gemini | MC7 | 6,5 | 2005 | 4,34 | 0 | 46 | 205 | 71068 | 9084 | 0 | 2 | 0,0 |
| Gemini | MC8 | 7,5 | 2004 | 4,41 | 1 | 49 | 214 | 73117 | 10422 | 0 | 2 | 0,1 |
| Gemini | MC9 | 8,5 | 2004 | 4,57 | 1 | 46 | 198 | 66309 | 10398 | 0 | 2 | 0,2 |
| Gemini | MC10 | 9,5 | 2003 | 4,62 | 2 | 62 | 223 | 71605 | 8345 | 0 | 2 | 0,3 |
| Gemini | MC11 | 10,5 | 2002 | 4,69 | 4 | 47 | 245 | 72133 | 10089 | 0 | 2 | 0,6 |
| Gemini | MC12 | 11,5 | 2002 | 4,55 | 2 | 41 | 203 | 61674 | 10229 | 0 | 2 | 0,4 |
| Gemini | MC13 | 12,5 | 2001 | 4,57 | 0 | 46 | 212 | 69192 | 10195 | 0 | 2 | 0,1 |
| Gemini | MC14 | 13,5 | 2001 | 4,69 | 1 | 50 | 228 | 77287 | 11036 | 0 | 2 | 0,2 |
| Gemini | MC15 | 14,5 | 2000 | 4,25 | 1 | 48 | 207 | 71944 | 11075 | 0 | 2 | 0,1 |
| Gemini | MC16 | 15,5 | 2000 | 4,21 | 0 | 44 | 194 | 65808 | 8931 | 0 | 1 | 0,0 |
| Gemini | MC17 | 16,5 | 1999 | 4,32 | 0 | 48 | 209 | 70097 | 9131 | 0 | 2 | 0,1 |
| Gemini | MC18 | 17,5 | 1999 | 4,35 | 1 | 51 | 226 | 75043 | 10338 | 0 | 2 | 0,2 |
| Gemini | MC19 | 18,5 | 1998 | 4,45 | 1 | 48 | 213 | 67880 | 9532 | 0 | 2 | 0,1 |
| Gemini | MC20 | 19,5 | 1997 | 4,49 | 2 | 50 | 229 | 72398 | 10464 | 0 | 2 | 0,3 |
| Gemini | MC21 | 20,5 | 1997 | 4,59 | 2 | 51 | 233 | 70732 | 11317 | 0 | 2 | 0,3 |
| Gemini | MC22 | 21,5 | 1996 | 4,64 | 2 | 51 | 239 | 67822 | 10842 | 0 | 2 | 0,4 |
| Gemini | MC23 | 22,5 | 1996 | 4,67 | 2 | 54 | 252 | 73522 | 11298 | 0 | 2 | 0,4 |
| Gemini | MC24 | 23,5 | 1995 | 4,61 | 3 | 52 | 246 | 72282 | 11686 | 0 | 2 | 0,5 |
| Gemini | MC25 | 24,5 | 1995 | 4,64 | 3 | 45 | 218 | 66075 | 10943 | 0 | 2 | 0,7 |
| Gemini | MC26 | 25,5 | 1994 | 4,44 | 3 | 56 | 230 | 72474 | 11479 | 0 | 3 | 0,5 |
| Gemini | MC27 | 26,5 | 1993 | 4,40 | 1 | 45 | 204 | 61780 | 9469 | 0 | 2 | 0,2 |
| Gemini | MC28 | 27,5 | 1993 | 4,45 | 0 | 52 | 227 | 68440 | 10117 | 0 | 2 | 0,1 |
| Gemini | MC29 | 28,5 | 1992 | 4,18 | 3 | 58 | 248 | 70595 | 11867 | 0 | 3 | 0,5 |
| Piston | LC1226 | 29,1 | 1992 | 4,44 | 5 | 48 | 248 | 71321 | 8708 | 0,12 | 2 | 0,9 |
| Gemini | MC30 | 29,5 | 1992 | 4,48 | 2 | 55 | 237 | 70713 | 10967 | 0 | 3 | 0,3 |
| Gemini | MC31 | 30,5 | 1991 | 4,41 | 0 | 55 | 228 | 70015 | 10806 | 0 | 2 | 0,1 |
| Gemini | MC32 | 31,5 | 1990 | 4,39 | 1 | 54 | 232 | 70934 | 9292 | 0 | 2 | 0,2 |
| Gemini | MC33 | 32,5 | 1989 | 4,45 | 1 | 58 | 243 | 71242 | 10879 | 0 | 2 | 0,2 |
| Gemini | MC34 | 33,5 | 1989 | 4,56 | 3 | 56 | 247 | 68974 | 11678 | 0 | 2 | 0,6 |
| Piston | LC1209 | 33,7 | 1989 | 4,57 | 6 | 53 | 258 | 71490 | 12443 | 0,15 | 3 | 0,7 |
| Gemini | MC35 | 34,5 | 1987 | 4,69 | 5 | 58 | 271 | 69982 | 11132 | 0 | 2 | 0,5 |
| Gemini | MC36 | 35,5 | 1984 | 4,72 | 7 | 57 | 281 | 69981 | 11255 | 0 | 3 | 0,4 |
| Gemini | MC37 | 36,5 | 1980 | 4,83 | 10 | 58 | 303 | 69305 | 12339 | 0 | 4 | 0,5 |
| Piston | LC1198 | 36,7 | 1976 | 4,69 | 13 | 59 | 335 | 69130 | 14400 | 0,04 | 7 | 0,2 |
| Gemini | MC38 | 37,5 | 1971 | 4,65 | 7 | 59 | 270 | 68613 | 11664 | 0 | 3 | 0,2 |
| Gemini | MC39 | 38,5 | 1965 | 4,59 | 5 | 64 | 267 | 71489 | 12731 | 0 | 3 | 0,2 |
| Gemini | MC40 | 39,5 | 1960 | 4,67 | 5 | 62 | 272 | 68611 | 12885 | 0 | 3 | 0,2 |
| Gemini | MC41 | 40,5 | 1960 | 4,71 | 6 | 62 | 292 | 70279 | 13340 | 0 | 4 | 0,3 |
| Piston | LC1184 | 40,5 | 1958 | 4,61 | 11 | 55 | 313 | 70252 | 13458 | 0,12 | 4 | 1,0 |
| Gemini | MC42 | 41,5 | 1958 | 4,80 | 11 | 60 | 286 | 68065 | 13695 | 0,02 | 9 | 1,0 |
| Gemini | MC43 | 42,5 | 1958 | 4,57 | 9 | 63 | 280 | 69689 | 13134 | 0 | 6 | 0,9 |
| Gemini | MC44 | 43,5 | 1958 | 4,55 | 7 | 56 | 270 | 68586 | 11862 | 0 | 3 | 0,6 |
| Gemini | MC45 | 44,5 | 1958 | 4,43 | 5 | 63 | 272 | 70416 | 11746 | 0 | 3 | 0,5 |
| Piston | LC1167 | 45,1 | 1958 | 4,48 | 9 | 54 | 282 | 71013 | 13948 | 0,13 | 3 | 0,8 |
| Gemini | MC46 | 45,5 | 1958 | 4,50 | 6 | 64 | 287 | 69507 | 13096 | 0 | 3 | 0,5 |
| Gemini | MC47 | 46,5 | 1958 | 4,48 | 5 | 63 | 283 | 71839 | 12812 | 0 | 3 | 0,4 |
| Gemini | MC48 | 47,5 | 1958 | 4,33 | 8 | 53 | 282 | 68649 | 12485 | 0,11 | 4 | 0,7 |
| Gemini | MC49 | 48,5 | 1958 | 4,30 | 3 | 47 | 264 | 68691 | 9859 | 0 | 3 | 0,3 |
| Piston | LC1154 | 48,6 | 1958 | 4,26 | 3 | 53 | 334 | 72813 | 12183 | 0,37 | 3 | 0,3 |
| Gemini | MC50 | 49,5 | 1958 | 4,34 | 0 | 61 | 265 | 69029 | 10022 | 0 | 3 | 0,0 |
| Gemini | MC51 | 50,5 | 1958 | 4,35 | 4 | 63 | 277 | 68225 | 10831 | 0 | 4 | 0,4 |
| Gemini | MC52 | 51,5 | 1958 | 4,18 | 7 | 62 | 279 | 67849 | 12884 | 0 | 9 | 0,6 |
| Piston | LC1140 | 52,1 | 1958 | 4,30 | 16 | 54 | 301 | 68182 | 11790 | 0,04 | 22 | 1,4 |
| Gemini | MC53 | 52,5 | 1958 | 4,06 | 2 | 58 | 254 | 69649 | 12259 | 0 | 4 | 0,2 |
| Gemini | MC54 | 53,5 | 1958 | 4,16 | 0 | 62 | 254 | 70336 | 9198 | 1,60 | 3 | 0,0 |
| Gemini | MC55 | 54,5 | 1958 | 4,09 | 0 | 60 | 252 | 71091 | 11543 | 0 | 3 | 0,0 |
| Gemini | MC56 | 55,5 | 1958 | 4,06 | 0 | 55 | 243 | 69298 | 10644 | 2,10 | 3 | 0,0 |
| Gemini | MC57 | 56,5 | 1958 | 3,89 | 0 | 60 | 247 | 70424 | 12225 | 4,40 | 3 | 0,0 |
| Piston | LC1122 | 56,8 | 1958 | 3,96 | 2 | 55 | 279 | 70832 | 12511 | 0,39 | 3 | 0,2 |
| Gemini | MC58 | 57,5 | 1958 | 4,01 | 0 | 61 | 245 | 71212 | 13047 | 0,66 | 3 | 0,0 |
| Gemini | MC59 | 58,5 | 1957 | 4,01 | 0 | 63 | 237 | 70244 | 11043 | 0,78 | 2 | 0,0 |
| Gemini | MC60 | 59,5 | 1957 | 4,02 | 0 | 63 | 242 | 71011 | 13902 | 1,43 | 3 | 0,0 |
| Gemini | MC61 | 60,5 | 1957 | 4,03 | 0 | 59 | 237 | 68623 | 12369 | 0,84 | 3 | 0,0 |
| Piston | LC1106 | 61,2 | 1957 | 4,07 | 3 | 57 | 243 | 74045 | 9082 | 0,23 | 3 | 0,6 |
| Gemini | MC62 | 61,5 | 1957 | 4,11 | 0 | 62 | 238 | 69393 | 10005 | 0,70 | 3 | 0,0 |
| Gemini | MC63 | 62,5 | 1957 | 4,20 | 0 | 64 | 245 | 70467 | 9107 | 0,59 | 3 | 0,0 |
| Piston | LC1101 | 62,5 | 1957 | 3,99 | 3 | 57 | 283 | 71010 | 9670 | 0,17 | 3 | 0,7 |
| Gemini | MC64 | 63,5 | 1957 | 4,17 | 0 | 64 | 243 | 72137 | 10357 | 0,90 | 3 | 0,0 |
| Piston | LC1096 | 63,9 | 1957 | 4,26 | 3 | 61 | 301 | 72869 | 7832 | 0,20 | 3 | 0,7 |
| Gemini | MC65 | 64,5 | 1957 | 4,26 | 0 | 61 | 250 | 70707 | 8912 | 0,75 | 3 | 0,0 |
| Piston | LC1091 | 66,0 | 1956 | 3,90 | 3 | 51 | 244 | 72500 | 7979 | 0,27 | 2 | 1,1 |
| Gemini | MC66 | 66,5 | 1955 | 4,18 | 0 | 65 | 248 | 71490 | 7995 | 0,93 | 5 | 0,0 |
| Piston | LC1087 | 67,8 | 1951 | 3,43 | 5 | 48 | 242 | 67886 | 18479 | 0,07 | 2 | 0,3 |
| Piston | LC1083 | 69,6 | 1943 | 3,72 | 7 | 51 | 272 | 71479 | 8443 | 0,07 | 2 | 0,3 |
| Piston | LC1078 | 71,9 | 1932 | 3,97 | 4 | 58 | 243 | 71382 | 6014 | 0,09 | 3 | 0,2 |
| Piston | LC1073 | 74,1 | 1923 | 3,61 | 4 | 52 | 222 | 74125 | 11653 | 0,15 | 2 | 0,2 |
| Piston | LC1069 | 75,9 | 1914 | 3,70 | 7 | 52 | 235 | 67907 | 18337 | 0,05 | 3 | 0,3 |
| Piston | LC1064 | 78,2 | 1904 | 3,72 | 2 | 54 | 220 | 71238 | 8073 | 0,12 | 4 | 0,1 |
| Piston | LC1060 | 80,0 | 1896 | 3,42 | 3 | 54 | 265 | 69646 | 11165 | 0,16 | 4 | 0,1 |
| Piston | LC1056 | 81,8 | 1888 | 3,46 | 1 | 56 | 210 | 71067 | 13765 | 0 | 4 | 0,0 |
| Piston | LC1051 | 84,0 | 1878 | 3,91 | 2 | 55 | 227 | 72831 | 5183 | 0,13 | 5 | 0,1 |
| Piston | LC1047 | 85,8 | 1870 | 3,83 | 3 | 53 | 215 | 73107 | 4806 | 0,09 | 4 | 0,1 |
| Piston | LC1043 | 87,6 | 1863 | 3,64 | 7 | 51 | 217 | 73006 | 8248 | 0,11 | 3 | 0,4 |
| Piston | LC1039 | 89,4 | 1854 | 3,41 | 7 | 49 | 211 | 67347 | 12802 | 0,05 | 4 | 0,3 |
| Piston | LC1035 | 91,2 | 1846 | 3,45 | 2 | 50 | 208 | 72878 | 6967 | 0,11 | 4 | 0,1 |
| Piston | LC1031 | 93,0 | 1839 | 3,52 | 2 | 54 | 208 | 71640 | 6832 | 0,14 | 5 | 0,1 |
| Piston | LC1027 | 94,8 | 1831 | 3,43 | 5 | 48 | 217 | 74205 | 6174 | 0,13 | 3 | 0,3 |
| Piston | LC1022 | 97,1 | 1821 | 3,32 | 3 | 50 | 179 | 72720 | 3994 | 0,01 | 3 | 0,2 |
| Piston | LC1017 | 99,3 | 1812 | 3,64 | 9 | 54 | 173 | 73737 | 4084 | 0,09 | 3 | 0,4 |
| Piston | LC1013 | 101,2 | 1804 | 3,38 | 2 | 50 | 203 | 74627 | 7109 | 0,40 | 6 | 0,1 |
| Piston | LC1009 | 103,2 | 1795 | 3,49 | 3 | 55 | 204 | 72626 | 7950 | 0,13 | 4 | 0,2 |
| Piston | LC1005 | 105,2 | 1787 | 3,50 | 3 | 53 | 199 | 73201 | 6671 | 0,14 | 4 | 0,2 |
| Piston | LC1001 | 107,1 | 1780 | 3,28 | 6 | 48 | 190 | 72044 | 8341 | 0,05 | 5 | 0,4 |
| Piston | LC998 | 108,6 | 1774 | 3,27 | 5 | 48 | 182 | 72625 | 8466 | 0,11 | 5 | 0,4 |
| Piston | LC993 | 111,1 | 1763 | 3,39 | 6 | 45 | 186 | 72913 | 7491 | 0,04 | 4 | 0,3 |
| Piston | LC988 | 113,5 | 1753 | 3,29 | 6 | 46 | 191 | 72911 | 9650 | 0,06 | 4 | 0,5 |
| Piston | LC984 | 115,5 | 1745 | 3,04 | 4 | 40 | 160 | 73905 | 10165 | 0,11 | 5 | 0,3 |
| Piston | LC980 | 117,5 | 1737 | 3,27 | 5 | 40 | 163 | 73742 | 7350 | 0,03 | 3 | 0,3 |
| Piston | LC976 | 119,5 | 1729 | 3,13 | 5 | 39 | 163 | 73932 | 7307 | 0,02 | 4 | 0,3 |
| Piston | LC973 | 120,9 | 1723 | 3,26 | 5 | 39 | 172 | 72617 | 10377 | 0,05 | 7 | 0,3 |
| Piston | LC969 | 122,9 | 1715 | 3,27 | 6 | 37 | 149 | 76117 | 6903 | 0,08 | 3 | 0,4 |
| Piston | LC965 | 124,9 | 1707 | 3,10 | 5 | 32 | 150 | 72839 | 12671 | 0,03 | 3 | 0,3 |
| Piston | LC961 | 126,8 | 1700 | 3,05 | 4 | 37 | 147 | 73348 | 11447 | 0,01 | 3 | 0,3 |
| Piston | LC957 | 128,8 | 1691 | 3,14 | 5 | 37 | 158 | 73849 | 10491 | 0 | 4 | 0,3 |
| Piston | LC953 | 130,8 | 1683 | 3,05 | 5 | 35 | 140 | 74309 | 13304 | 0,08 | 5 | 0,3 |
| Piston | LC949 | 132,8 | 1675 | 3,25 | 7 | 32 | 152 | 74351 | 7409 | 0,01 | 4 | 0,5 |
| Piston | LC945 | 134,7 | 1667 | 2,90 | 7 | 26 | 145 | 71824 | 15087 | 0,01 | 3 | 0,4 |
| Piston | LC941 | 136,7 | 1659 | 3,17 | 4 | 27 | 147 | 76152 | 5222 | 0,02 | 3 | 0,3 |
| Piston | LC938 | 138,2 | 1653 | 3,04 | 4 | 35 | 146 | 76743 | 10800 | 0,10 | 3 | 0,3 |
| Piston | LC934 | 140,1 | 1646 | 3,00 | 6 | 27 | 145 | 73045 | 12280 | 0,01 | 3 | 0,4 |
| Piston | LC930 | 142,1 | 1638 | 3,00 | 7 | 34 | 152 | 72583 | 14484 | 0,01 | 3 | 0,4 |
| Piston | LC925 | 144,6 | 1628 | 3,03 | 5 | 33 | 145 | 75471 | 12762 | 0,04 | 3 | 0,4 |
| Piston | LC921 | 146,6 | 1620 | 3,18 | 5 | 36 | 144 | 75229 | 7631 | 0 | 3 | 0,4 |
| Piston | LC918 | 148,0 | 1614 | 3,14 | 5 | 33 | 144 | 74186 | 10171 | 0 | 3 | 0,3 |
| Piston | LC914 | 150,0 | 1606 | 3,15 | 5 | 34 | 140 | 72715 | 8622 | 0,05 | 2 | 0,3 |
| Piston | LC911 | 151,5 | 1600 | 3,13 | 6 | 32 | 131 | 75100 | 8785 | 0,06 | 3 | 0,4 |
| Piston | LC910 | 152,0 | 1598 | 3,14 | 3 | 33 | 139 | 70909 | 8284 | 0,08 | 2 | 0,2 |
| Piston | LC907 | 153,4 | 1592 | 3,11 | 4 | 33 | 131 | 71804 | 7671 | 0,05 | 3 | 0,3 |
| Piston | LC903 | 155,4 | 1584 | 3,16 | 4 | 32 | 138 | 71933 | 13141 | 0,05 | 3 | 0,2 |
| Piston | LC900 | 156,9 | 1578 | 3,42 | 5 | 35 | 138 | 70632 | 10993 | 0,05 | 3 | 0,3 |
| Piston | LC896 | 158,9 | 1570 | 3,23 | 6 | 33 | 131 | 68714 | 12249 | 0,04 | 3 | 0,4 |
| Piston | LC894 | 159,9 | 1566 | 3,45 | 8 | 32 | 135 | 75105 | 9895 | 0,04 | 3 | 0,5 |
| Piston | LC893 | 160,3 | 1565 | 3,48 | 6 | 35 | 139 | 72192 | 10591 | 0,04 | 3 | 0,6 |
| Piston | LC889 | 162,3 | 1557 | 3,60 | 7 | 35 | 142 | 70067 | 10227 | 0,13 | 3 | 0,5 |
| Piston | LC886 | 163,8 | 1551 | 3,56 | 10 | 32 | 143 | 71747 | 10836 | 0,03 | 3 | 0,7 |
| Piston | LC882 | 165,8 | 1544 | 3,57 | 7 | 33 | 145 | 71326 | 10455 | 0,04 | 4 | 0,5 |
| Piston | LC881 | 166,3 | 1542 | 3,64 | 9 | 33 | 149 | 75825 | 11151 | 0,05 | 3 | 0,7 |
| Piston | LC879 | 167,2 | 1538 | 3,56 | 12 | 33 | 140 | 71561 | 12193 | 0,02 | 4 | 0,9 |
| Piston | LC875 | 169,2 | 1530 | 3,58 | 10 | 32 | 137 | 70756 | 10506 | 0,03 | 3 | 0,8 |
| Piston | LC872 | 170,7 | 1524 | 3,60 | 8 | 40 | 141 | 72103 | 12146 | 0,03 | 3 | 0,6 |
| Piston | LC868 | 172,7 | 1516 | 3,62 | 10 | 33 | 140 | 71714 | 13880 | 0,03 | 3 | 0,7 |
| Piston | LC865 | 174,1 | 1511 | 3,68 | 11 | 34 | 143 | 72578 | 10576 | 0,03 | 3 | 0,8 |
| Piston | LC862 | 175,6 | 1505 | 3,67 | 10 | 31 | 138 | 75711 | 7653 | 0,04 | 4 | 0,8 |
| Piston | LC861 | 176,1 | 1503 | 3,68 | 10 | 35 | 136 | 70593 | 19618 | 0,03 | 4 | 0,8 |
| Piston | LC858 | 177,6 | 1498 | 3,65 | 11 | 33 | 135 | 70605 | 12101 | 0,02 | 3 | 0,9 |
| Piston | LC854 | 179,6 | 1490 | 3,69 | 9 | 33 | 134 | 71139 | 8813 | 0,03 | 3 | 0,7 |
| Piston | LC851 | 181,0 | 1484 | 3,74 | 19 | 36 | 141 | 72262 | 13174 | 0,02 | 3 | 1,3 |
| Piston | LC847 | 183,0 | 1477 | 3,72 | 13 | 32 | 133 | 74087 | 11744 | 0,04 | 3 | 1,0 |
| Piston | LC844 | 184,5 | 1471 | 3,81 | 12 | 33 | 136 | 69200 | 11206 | 0,03 | 3 | 0,9 |
| Piston | LC840 | 186,5 | 1463 | 3,84 | 8 | 34 | 135 | 70598 | 11678 | 0,03 | 3 | 0,6 |
| Piston | LC837 | 187,9 | 1458 | 4,03 | 13 | 37 | 139 | 71434 | 11225 | 0,03 | 3 | 1,2 |
| Piston | LC833 | 189,9 | 1450 | 4,11 | 15 | 36 | 142 | 71220 | 10375 | 0,03 | 3 | 1,0 |
| Piston | LC831 | 190,9 | 1446 | 4,00 | 12 | 36 | 137 | 71213 | 11223 | 0,03 | 3 | 0,9 |
| Piston | LC830 | 191,4 | 1444 | 4,00 | 15 | 33 | 131 | 70821 | 10523 | 0,03 | 2 | 1,2 |
| Piston | LC829 | 191,9 | 1443 | 4,04 | 17 | 32 | 135 | 68830 | 11438 | 0,02 | 3 | 1,6 |
| Piston | LC826 | 193,3 | 1438 | 4,24 | 11 | 34 | 137 | 69757 | 12539 | 0,03 | 4 | 1,0 |
| Piston | LC823 | 194,8 | 1432 | 4,12 | 6 | 31 | 129 | 68483 | 9290 | 0,07 | 7 | 0,4 |
| Piston | LC819 | 196,8 | 1425 | 4,32 | 5 | 31 | 134 | 71393 | 7789 | 0,06 | 3 | 0,5 |
| Piston | LC817 | 197,8 | 1421 | 4,09 | 12 | 34 | 134 | 69675 | 11726 | 0,03 | 3 | 0,9 |
| Piston | LC816 | 198,3 | 1419 | 4,07 | 14 | 33 | 138 | 70501 | 10540 | 0,03 | 2 | 1,0 |
| Piston | LC814 | 199,3 | 1415 | 4,20 | 11 | 33 | 135 | 69720 | 6897 | 0,03 | 3 | 0,8 |
| Piston | LC812 | 200,3 | 1412 | 4,38 | 12 | 30 | 134 | 70325 | 7060 | 0,08 | 6 | 1,2 |
| Piston | LC811 | 200,8 | 1410 | 4,30 | 7 | 32 | 134 | 67758 | 8587 | 0,05 | 20 | 0,7 |
| Piston | LC809 | 201,8 | 1406 | 4,34 | 23 | 36 | 145 | 69138 | 18303 | 0,02 | 5 | 1,8 |
| Piston | LC807 | 202,8 | 1402 | 4,33 | 12 | 32 | 134 | 67389 | 15614 | 0,03 | 11 | 0,9 |
| Piston | LC805 | 203,8 | 1399 | 4,31 | 18 | 31 | 135 | 66181 | 18420 | 0,02 | 19 | 1,6 |
| Piston | LC802 | 205,3 | 1393 | 4,22 | 11 | 32 | 136 | 71098 | 8303 | 0,07 | 3 | 0,9 |
| Piston | LC800 | 206,3 | 1389 | 4,18 | 14 | 30 | 135 | 72102 | 16991 | 0,03 | 4 | 1,1 |
| Piston | LC798 | 207,3 | 1386 | 4,06 | 20 | 32 | 142 | 68519 | 18251 | 0,02 | 15 | 2,0 |
| Piston | LC795 | 208,8 | 1380 | 3,95 | 15 | 30 | 129 | 67975 | 13598 | 0,02 | 13 | 1,2 |
| Piston | LC793 | 209,8 | 1376 | 4,13 | 11 | 30 | 125 | 67975 | 10899 | 0,03 | 18 | 0,8 |
| Piston | LC791 | 210,8 | 1372 | 4,17 | 14 | 27 | 127 | 68042 | 12551 | 0,02 | 13 | 1,2 |
| Piston | LC788 | 212,3 | 1367 | 4,27 | 13 | 28 | 129 | 67541 | 11758 | 0,02 | 23 | 1,2 |
| Piston | LC786 | 213,3 | 1363 | 4,22 | 19 | 31 | 131 | 65967 | 16059 | 0,02 | 26 | 1,4 |
| Piston | LC784 | 214,3 | 1359 | 4,09 | 11 | 31 | 135 | 69482 | 12428 | 0,04 | 11 | 0,8 |
| Piston | LC782 | 215,3 | 1355 | 4,21 | 16 | 28 | 130 | 69534 | 16045 | 0 | 11 | 1,2 |
| Piston | LC781 | 215,8 | 1353 | 4,16 | 12 | 29 | 134 | 71851 | 10606 | 0,03 | 13 | 1,1 |
| Piston | LC779 | 216,8 | 1350 | 4,05 | 22 | 28 | 132 | 65943 | 22253 | 0,03 | 44 | 2,1 |
| Piston | LC777 | 217,8 | 1346 | 4,09 | 12 | 29 | 131 | 68792 | 12011 | 0,04 | 7 | 0,9 |
| Piston | LC774 | 219,3 | 1340 | 4,11 | 16 | 30 | 132 | 66849 | 16392 | 0,03 | 16 | 1,2 |
| Piston | LC772 | 220,3 | 1337 | 3,95 | 8 | 28 | 129 | 68809 | 11265 | 0,05 | 19 | 0,7 |
| Piston | LC770 | 221,3 | 1333 | 3,75 | 13 | 29 | 133 | 70822 | 16071 | 0,04 | 6 | 1,1 |
| Piston | LC767 | 222,8 | 1327 | 3,51 | 10 | 30 | 133 | 74731 | 12340 | 0,04 | 3 | 0,9 |
| Piston | LC765 | 223,8 | 1323 | 3,45 | 10 | 30 | 132 | 71117 | 17644 | 0,03 | 5 | 0,8 |
| Piston | LC763 | 224,8 | 1320 | 3,55 | 8 | 28 | 131 | 73295 | 10110 | 0,01 | 3 | 0,8 |
| Piston | LC760 | 226,3 | 1314 | 3,44 | 7 | 26 | 131 | 73536 | 11706 | 0,03 | 3 | 0,6 |
| Piston | LC758 | 227,3 | 1310 | 3,53 | 10 | 30 | 131 | 72115 | 11209 | 0,03 | 4 | 0,8 |
| Piston | LC756 | 228,3 | 1306 | 3,46 | 7 | 27 | 128 | 72838 | 9442 | 0,03 | 4 | 0,6 |
| Piston | LC753 | 229,8 | 1301 | 3,56 | 6 | 29 | 129 | 71652 | 10693 | 0,04 | 4 | 0,6 |
| Piston | LC750 | 231,3 | 1295 | 3,63 | 11 | 29 | 128 | 74060 | 9807 | 0,03 | 3 | 0,9 |
| Piston | LC749 | 231,8 | 1293 | 3,62 | 11 | 32 | 135 | 71228 | 15012 | 0,03 | 5 | 0,9 |
| Piston | LC746 | 233,3 | 1287 | 3,56 | 7 | 29 | 129 | 70914 | 11213 | 0,03 | 5 | 0,6 |
| Piston | LC744 | 234,3 | 1283 | 3,53 | 11 | 31 | 129 | 71679 | 13511 | 0,02 | 5 | 0,9 |
| Piston | LC742 | 235,3 | 1280 | 3,58 | 13 | 31 | 131 | 71198 | 17797 | 0,02 | 5 | 1,3 |
| Piston | LC739 | 236,8 | 1274 | 3,60 | 13 | 28 | 130 | 72054 | 17377 | 0,02 | 4 | 1,2 |
| Piston | LC737 | 237,8 | 1270 | 3,63 | 13 | 30 | 131 | 71418 | 14576 | 0,02 | 7 | 1,0 |
| Piston | LC735 | 238,8 | 1266 | 3,55 | 9 | 30 | 125 | 72890 | 12870 | 0,04 | 6 | 0,8 |
| Piston | LC732 | 240,3 | 1260 | 3,66 | 8 | 27 | 127 | 73568 | 7693 | 0,03 | 6 | 0,6 |
| Piston | LC730 | 241,3 | 1257 | 3,75 | 11 | 31 | 135 | 72282 | 9268 | 0 | 8 | 1,2 |
| Piston | LC728 | 242,3 | 1253 | 3,96 | 14 | 32 | 125 | 69874 | 8754 | 0,02 | 3 | 1,1 |
| Piston | LC725 | 243,8 | 1247 | 4,16 | 13 | 35 | 134 | 69202 | 14311 | 0,09 | 5 | 1,0 |
| Piston | LC721 | 245,8 | 1239 | 4,12 | 13 | 33 | 135 | 70359 | 14829 | 0,02 | 6 | 1,1 |
| Piston | LC720 | 246,3 | 1237 | 4,13 | 10 | 32 | 137 | 74165 | 11327 | 0,04 | 5 | 0,8 |
| Piston | LC718 | 247,3 | 1233 | 4,13 | 12 | 30 | 134 | 67904 | 11993 | 0,02 | 12 | 0,9 |
| Piston | LC715 | 248,8 | 1227 | 4,36 | 11 | 30 | 132 | 68104 | 10853 | 0,02 | 13 | 0,9 |
| Piston | LC712 | 250,3 | 1221 | 4,59 | 19 | 33 | 143 | 68908 | 12890 | 0,02 | 7 | 1,5 |
| Piston | LC710 | 251,3 | 1217 | 4,75 | 16 | 32 | 148 | 69758 | 7171 | 0 | 21 | 1,1 |
| Piston | LC708 | 252,3 | 1214 | 4,43 | 13 | 32 | 132 | 68323 | 7499 | 0,06 | 38 | 1,2 |
| Piston | LC705 | 253,8 | 1208 | 4,79 | 20 | 32 | 134 | 67383 | 16902 | 0,01 | 6 | 1,7 |
| Piston | LC702 | 255,3 | 1202 | 4,74 | 19 | 30 | 138 | 72572 | 11453 | 0,02 | 4 | 1,4 |
| Piston | LC701 | 255,8 | 1200 | 4,76 | 17 | 31 | 134 | 69635 | 9344 | 0,02 | 4 | 1,4 |
| Piston | LC698 | 257,3 | 1194 | 4,51 | 12 | 30 | 128 | 68045 | 12855 | 0,02 | 25 | 1,0 |
| Piston | LC696 | 258,3 | 1190 | 4,66 | 14 | 28 | 133 | 70415 | 12409 | 0 | 6 | 1,0 |
| Piston | LC694 | 259,3 | 1186 | 4,44 | 14 | 32 | 130 | 70768 | 11396 | 0,02 | 4 | 1,1 |
| Piston | LC691 | 260,8 | 1180 | 4,20 | 9 | 32 | 131 | 68907 | 15230 | 0,03 | 5 | 0,7 |
| Piston | LC689 | 261,8 | 1175 | 4,45 | 11 | 28 | 134 | 70619 | 13039 | 0 | 3 | 0,7 |
| Piston | LC687 | 262,8 | 1171 | 4,53 | 5 | 29 | 126 | 72126 | 8680 | 0 | 3 | 0,4 |
| Piston | LC684 | 264,3 | 1165 | 4,46 | 12 | 29 | 129 | 70359 | 8574 | 0,02 | 6 | 0,9 |
| Piston | LC681 | 265,8 | 1159 | 4,31 | 6 | 30 | 132 | 68311 | 10397 | 0,04 | 5 | 0,5 |
| Piston | LC678 | 267,3 | 1153 | 4,50 | 11 | 32 | 136 | 71597 | 10250 | 0 | 3 | 0,8 |
| Piston | LC676 | 268,3 | 1148 | 4,50 | 9 | 29 | 130 | 68507 | 10068 | 0,02 | 3 | 0,6 |
| Piston | LC674 | 269,3 | 1144 | 4,43 | 3 | 32 | 128 | 69560 | 6675 | 0,07 | 5 | 0,2 |
| Piston | LC672 | 270,3 | 1140 | 4,50 | 12 | 32 | 137 | 70114 | 7336 | 0,06 | 4 | 0,9 |
| Piston | LC670 | 271,3 | 1136 | 4,39 | 13 | 32 | 127 | 67671 | 10040 | 0,02 | 9 | 1,0 |
| Piston | LC668 | 272,3 | 1132 | 4,55 | 11 | 30 | 129 | 70447 | 10730 | 0,02 | 11 | 0,9 |
| Piston | LC667 | 272,8 | 1130 | 4,56 | 12 | 31 | 130 | 67761 | 10397 | 0,02 | 12 | 0,8 |
| Piston | LC665 | 273,8 | 1126 | 4,66 | 8 | 30 | 130 | 71001 | 7967 | 0 | 3 | 0,6 |
| Piston | LC663 | 274,8 | 1121 | 4,55 | 10 | 30 | 129 | 67205 | 14255 | 0,03 | 4 | 0,7 |
| Piston | LC660 | 276,3 | 1115 | 4,56 | 8 | 30 | 147 | 67382 | 15275 | 0,09 | 10 | 0,6 |
| Piston | LC658 | 277,3 | 1111 | 4,78 | 9 | 27 | 133 | 71261 | 7535 | 0 | 5 | 0,7 |
| Piston | LC656 | 278,3 | 1107 | 4,79 | 15 | 30 | 125 | 66931 | 13329 | 0,02 | 5 | 1,1 |
| Piston | LC653 | 279,8 | 1101 | 4,93 | 13 | 30 | 129 | 67788 | 12237 | 0,02 | 12 | 1,0 |
| Piston | LC652 | 280,3 | 1099 | 5,06 | 12 | 28 | 124 | 68443 | 13137 | 0,03 | 19 | 1,0 |
| Piston | LC651 | 280,8 | 1097 | 5,10 | 17 | 30 | 133 | 66339 | 16326 | 0,02 | 6 | 1,0 |
| Piston | LC649 | 281,8 | 1092 | 5,10 | 15 | 32 | 133 | 68434 | 10629 | 0,03 | 3 | 0,9 |
| Piston | LC646 | 283,3 | 1086 | 5,26 | 9 | 30 | 129 | 67341 | 11397 | 0,04 | 2 | 0,7 |
| Piston | LC644 | 284,3 | 1082 | 5,28 | 11 | 28 | 133 | 67844 | 15052 | 0,03 | 4 | 0,9 |
| Piston | LC642 | 285,3 | 1078 | 5,39 | 16 | 29 | 134 | 67731 | 14411 | 0,02 | 4 | 1,2 |
| Piston | LC639 | 286,8 | 1072 | 5,02 | 13 | 28 | 128 | 68569 | 12549 | 0,03 | 3 | 1,0 |
| Piston | LC637 | 287,8 | 1068 | 5,09 | 17 | 29 | 131 | 67755 | 14965 | 0,02 | 4 | 1,4 |
| Piston | LC635 | 288,8 | 1064 | 5,26 | 13 | 23 | 128 | 70574 | 11939 | 0,04 | 3 | 1,0 |
| Piston | LC634 | 289,3 | 1062 | 5,05 | 13 | 26 | 126 | 65250 | 17555 | 0,03 | 27 | 1,0 |
| Piston | LC632 | 290,3 | 1058 | 4,94 | 14 | 28 | 126 | 66545 | 14908 | 0,02 | 18 | 1,1 |
| Piston | LC628 | 292,3 | 1050 | 5,19 | 15 | 28 | 131 | 68509 | 14584 | 0,02 | 12 | 1,3 |
| Piston | LC625 | 293,8 | 1044 | 5,19 | 17 | 27 | 129 | 68368 | 10638 | 0,02 | 4 | 1,3 |
| Piston | LC624 | 294,3 | 1042 | 5,22 | 14 | 24 | 146 | 70106 | 8869 | 0 | 4 | 1,1 |
| Piston | LC621 | 295,8 | 1036 | 5,37 | 20 | 28 | 130 | 68020 | 12470 | 0,02 | 5 | 1,4 |
| Piston | LC619 | 296,8 | 1032 | 5,17 | 10 | 27 | 134 | 67937 | 12845 | 0 | 19 | 0,8 |
| Piston | LC618 | 297,3 | 1030 | 5,12 | 17 | 26 | 122 | 65073 | 19631 | 0,02 | 19 | 1,4 |
| Piston | LC616 | 298,3 | 1025 | 5,01 | 17 | 21 | 136 | 68319 | 13802 | 0 | 13 | 1,1 |
| Piston | LC614 | 299,3 | 1019 | 4,92 | 8 | 27 | 121 | 65111 | 8325 | 0,03 | 20 | 0,5 |
| Piston | LC611 | 300,7 | 1005 | 4,97 | 24 | 27 | 125 | 63314 | 12817 | 0,02 | 22 | 0,8 |
| Piston | LC609 | 301,7 | 994 | 5,30 | 20 | 22 | 144 | 71424 | 8796 | 0 | 6 | 0,5 |
| Piston | LC607 | 302,7 | 983 | 5,28 | 22 | 30 | 131 | 68259 | 10536 | 0,02 | 6 | 0,6 |
| Piston | LC604 | 304,1 | 968 | 5,30 | 24 | 26 | 133 | 66363 | 13668 | 0,02 | 11 | 0,7 |
| Piston | LC602 | 305,1 | 958 | 5,05 | 16 | 24 | 143 | 69120 | 16591 | 0,01 | 8 | 0,5 |
| Piston | LC600 | 306,1 | 947 | 5,02 | 14 | 25 | 131 | 68647 | 12442 | 0,02 | 28 | 0,4 |
| Piston | LC597 | 307,5 | 933 | 5,09 | 23 | 25 | 128 | 64304 | 13104 | 0,01 | 30 | 0,7 |
| Piston | LC593 | 309,5 | 911 | 5,28 | 27 | 27 | 132 | 66259 | 22139 | 0,01 | 5 | 0,8 |
| Piston | LC590 | 310,9 | 896 | 5,05 | 18 | 26 | 129 | 66116 | 18149 | 0,02 | 4 | 0,6 |
| Piston | LC588 | 311,9 | 885 | 4,80 | 13 | 24 | 145 | 71040 | 13993 | 0,01 | 4 | 0,4 |
| Piston | LC586 | 312,9 | 874 | 4,68 | 28 | 27 | 132 | 67182 | 17538 | 0,02 | 12 | 0,8 |
| Piston | LC583 | 314,3 | 859 | 4,81 | 25 | 26 | 123 | 64427 | 16334 | 0,02 | 31 | 0,8 |
| Piston | LC581 | 315,3 | 848 | 4,79 | 17 | 25 | 138 | 72693 | 9115 | 0,03 | 11 | 0,5 |
| Piston | LC578 | 316,7 | 834 | 4,87 | 28 | 25 | 125 | 65036 | 22995 | 0,01 | 22 | 0,9 |
| Piston | LC576 | 317,7 | 823 | 4,79 | 26 | 28 | 130 | 67268 | 14122 | 0,02 | 20 | 0,8 |
| Piston | LC574 | 318,7 | 812 | 4,85 | 19 | 21 | 146 | 69577 | 14820 | 0,01 | 10 | 0,6 |
| Piston | LC572 | 319,7 | 801 | 4,77 | 17 | 25 | 123 | 66762 | 17707 | 0,02 | 23 | 0,5 |
| Piston | LC569 | 321,1 | 786 | 4,37 | 44 | 25 | 122 | 61370 | 14861 | 0,03 | 76 | 1,5 |
| Piston | LC567 | 322,1 | 775 | 4,37 | 35 | 20 | 139 | 69509 | 16484 | 0 | 18 | 1,0 |
| Piston | LC565 | 323,1 | 763 | 4,30 | 19 | 23 | 126 | 67892 | 16850 | 0,02 | 9 | 0,5 |
| Piston | LC563 | 324,0 | 752 | 4,54 | 15 | 24 | 132 | 71342 | 10703 | 0,03 | 3 | 0,4 |
| Piston | LC562 | 324,5 | 746 | 4,75 | 14 | 24 | 126 | 68903 | 9067 | 0,02 | 3 | 0,4 |
| Piston | LC559 | 326,0 | 727 | 4,83 | 22 | 26 | 129 | 67503 | 18283 | 0,02 | 4 | 0,5 |
| Piston | LC557 | 326,9 | 716 | 5,07 | 19 | 23 | 139 | 69322 | 14949 | 0,01 | 7 | 0,5 |
| Piston | LC555 | 327,9 | 705 | 5,09 | 29 | 26 | 124 | 66334 | 17756 | 0,01 | 4 | 0,8 |
| Piston | LC552 | 329,4 | 691 | 5,46 | 29 | 25 | 127 | 66170 | 15629 | 0,01 | 4 | 1,0 |
| Piston | LC550 | 330,3 | 683 | 5,45 | 27 | 23 | 139 | 68726 | 11444 | 0,01 | 3 | 1,0 |
| Piston | LC548 | 331,3 | 674 | 5,22 | 24 | 25 | 129 | 65748 | 18281 | 0,02 | 5 | 0,8 |
| Piston | LC547 | 331,8 | 670 | 5,21 | 25 | 25 | 135 | 68916 | 22782 | 0,02 | 4 | 1,0 |
| Piston | LC545 | 332,8 | 662 | 5,22 | 20 | 24 | 122 | 65906 | 11871 | 0,02 | 25 | 0,9 |
| Piston | LC541 | 334,7 | 646 | 5,24 | 26 | 24 | 121 | 62803 | 15534 | 0,01 | 29 | 1,1 |
| Piston | LC538 | 336,2 | 634 | 5,33 | 13 | 26 | 124 | 66798 | 17265 | 0,02 | 4 | 0,5 |
| Piston | LC534 | 338,1 | 619 | 5,22 | 19 | 26 | 123 | 65583 | 15052 | 0,02 | 11 | 0,9 |
| Piston | LC531 | 339,6 | 607 | 5,09 | 23 | 21 | 122 | 62455 | 17604 | 0 | 56 | 0,9 |
| Piston | LC530 | 340,0 | 604 | 5,20 | 18 | 25 | 124 | 64021 | 16766 | 0,02 | 25 | 0,9 |
| Piston | LC527 | 341,5 | 592 | 5,36 | 14 | 24 | 127 | 65694 | 14376 | 0,03 | 3 | 0,5 |
| Piston | LC524 | 343,0 | 580 | 5,19 | 17 | 26 | 128 | 68593 | 11335 | 0,02 | 5 | 0,6 |
| Piston | LC522 | 343,9 | 573 | 5,24 | 20 | 25 | 139 | 67791 | 13005 | 0 | 20 | 0,8 |
| Piston | LC520 | 344,9 | 565 | 5,36 | 17 | 24 | 132 | 67316 | 11569 | 0,02 | 4 | 0,6 |
| Piston | LC517 | 346,4 | 553 | 5,20 | 16 | 24 | 131 | 67524 | 11179 | 0,02 | 4 | 0,6 |
| Piston | LC515 | 347,3 | 546 | 5,00 | 12 | 26 | 124 | 66325 | 12164 | 0,03 | 12 | 0,5 |
| Piston | LC513 | 348,3 | 538 | 4,99 | 20 | 26 | 130 | 68413 | 15828 | 0,02 | 5 | 0,7 |
| Piston | LC512 | 348,8 | 534 | 4,90 | 22 | 27 | 136 | 69877 | 20077 | 0,02 | 5 | 0,9 |
| Piston | LC510 | 349,8 | 526 | 5,00 | 9 | 24 | 125 | 66610 | 10697 | 0,04 | 8 | 0,4 |
| Piston | LC508 | 350,7 | 518 | 5,10 | 15 | 24 | 145 | 69597 | 13320 | 0 | 6 | 0,7 |
| Piston | LC506 | 351,2 | 514 | 5,14 | 14 | 26 | 131 | 68071 | 10976 | 0,03 | 4 | 0,5 |
| Piston | LC503 | 351,7 | 509 | 5,17 | 15 | 26 | 129 | 67286 | 10676 | 0,03 | 9 | 0,5 |
| Piston | LC501 | 353,2 | 497 | 5,01 | 16 | 25 | 125 | 63355 | 17308 | 0,02 | 30 | 0,6 |
| Piston | LC499 | 354,1 | 490 | 5,11 | 18 | 25 | 128 | 67545 | 10974 | 0,02 | 5 | 0,8 |
| Piston | LC497 | 355,1 | 482 | 5,10 | 13 | 26 | 135 | 68817 | 13291 | 0,03 | 4 | 0,5 |
| Piston | LC496 | 356,6 | 470 | 5,15 | 21 | 25 | 135 | 69033 | 14205 | 0,02 | 5 | 0,8 |
| Piston | LC494 | 357,5 | 462 | 4,96 | 21 | 24 | 129 | 67070 | 13165 | 0,02 | 13 | 0,9 |
| Piston | LC492 | 358,5 | 454 | 4,74 | 13 | 25 | 129 | 67384 | 10365 | 0,03 | 32 | 0,5 |
| Piston | LC489 | 360,0 | 442 | 4,58 | 16 | 24 | 125 | 62004 | 15725 | 0,06 | 69 | 0,7 |
| Piston | LC487 | 360,9 | 434 | 4,96 | 27 | 25 | 166 | 67924 | 10778 | 0,02 | 8 | 1,1 |
| Piston | LC484 | 362,4 | 422 | 4,73 | 20 | 30 | 138 | 69767 | 12972 | 0,09 | 4 | 0,8 |
| Piston | LC482 | 363,3 | 414 | 4,65 | 19 | 27 | 133 | 66811 | 23040 | 0,02 | 9 | 0,8 |
| Piston | LC481 | 363,8 | 410 | 4,68 | 15 | 26 | 126 | 67350 | 13221 | 0,03 | 17 | 0,6 |
| Piston | LC480 | 364,3 | 406 | 4,52 | 14 | 27 | 128 | 68717 | 10172 | 0,03 | 27 | 0,6 |
| Piston | LC479 | 364,8 | 402 | 4,63 | 19 | 26 | 122 | 65426 | 11976 | 0,02 | 15 | 0,8 |
| Piston | LC477 | 365,8 | 393 | 4,73 | 12 | 26 | 129 | 69358 | 8248 | 0,03 | 11 | 0,5 |
| Piston | LC474 | 367,2 | 381 | 4,64 | 22 | 27 | 128 | 66147 | 16748 | 0,02 | 12 | 1,0 |
| Piston | LC471 | 368,7 | 368 | 4,54 | 8 | 25 | 135 | 69671 | 10437 | 0,01 | 6 | 0,3 |
| Piston | LC470 | 369,2 | 364 | 4,51 | 10 | 28 | 145 | 69076 | 11576 | 0,04 | 6 | 0,4 |
| Piston | LC467 | 370,6 | 352 | 4,47 | 29 | 27 | 128 | 64407 | 20863 | 0,01 | 21 | 1,1 |
| Piston | LC466 | 371,1 | 348 | 4,66 | 19 | 22 | 121 | 64180 | 13703 | 0,03 | 14 | 0,7 |
| Piston | LC463 | 372,6 | 334 | 4,61 | 15 | 30 | 128 | 67841 | 14531 | 0,03 | 7 | 0,6 |
| Piston | LC460 | 374,0 | 323 | 4,76 | 20 | 27 | 130 | 68113 | 16354 | 0,02 | 4 | 0,6 |
| Piston | LC458 | 375,0 | 314 | 4,82 | 21 | 29 | 139 | 67800 | 16148 | 0,01 | 7 | 0,8 |
| Piston | LC456 | 376,0 | 305 | 4,86 | 22 | 28 | 132 | 67969 | 14892 | 0,02 | 3 | 0,8 |
| Piston | LC453 | 377,4 | 292 | 4,80 | 21 | 27 | 133 | 68196 | 18316 | 0,03 | 3 | 0,8 |
| Piston | LC450 | 378,9 | 279 | 4,81 | 20 | 24 | 134 | 70761 | 14940 | 0,03 | 4 | 0,8 |
| Piston | LC449 | 379,4 | 275 | 4,88 | 19 | 27 | 130 | 68530 | 17796 | 0,03 | 3 | 0,8 |
| Piston | LC446 | 380,8 | 262 | 5,28 | 15 | 27 | 137 | 65827 | 10228 | 0,04 | 2 | 0,6 |
| Piston | LC444 | 381,8 | 253 | 5,38 | 19 | 21 | 140 | 68490 | 16012 | 0,02 | 3 | 0,6 |
| Piston | LC442 | 382,8 | 245 | 5,18 | 19 | 25 | 129 | 66937 | 15810 | 0,03 | 4 | 0,7 |
| Piston | LC439 | 384,2 | 233 | 5,17 | 18 | 26 | 127 | 65022 | 17927 | 0,03 | 18 | 0,7 |
| Piston | LC437 | 385,2 | 224 | 5,13 | 26 | 26 | 137 | 65781 | 22408 | 0,01 | 25 | 1,0 |
| Piston | LC435 | 386,2 | 216 | 5,14 | 28 | 25 | 126 | 63536 | 20665 | 0,02 | 41 | 1,0 |
| Piston | LC433 | 387,1 | 208 | 5,16 | 29 | 26 | 125 | 62978 | 15188 | 0,02 | 25 | 1,1 |
| Piston | LC432 | 387,6 | 203 | 5,36 | 30 | 25 | 129 | 67144 | 17385 | 0 | 13 | 1,0 |
| Piston | LC430 | 388,6 | 195 | 5,14 | 22 | 26 | 148 | 66066 | 20041 | 0,03 | 4 | 0,8 |
| Piston | LC428 | 389,6 | 187 | 5,18 | 26 | 28 | 134 | 64038 | 23925 | 0,02 | 30 | 1,1 |
| Piston | LC425 | 391,0 | 175 | 5,08 | 20 | 25 | 130 | 65916 | 16193 | 0,02 | 14 | 0,8 |
| Piston | LC423 | 392,0 | 167 | 4,87 | 18 | 28 | 137 | 68193 | 11514 | 0 | 15 | 0,7 |
| Piston | LC421 | 393,0 | 159 | 5,06 | 32 | 26 | 133 | 67553 | 21777 | 0,01 | 12 | 1,2 |
| Piston | LC418 | 394,4 | 148 | 5,21 | 18 | 26 | 130 | 68010 | 14074 | 0,03 | 7 | 0,7 |
| Piston | LC416 | 395,4 | 140 | 4,97 | 24 | 25 | 131 | 68159 | 14708 | 0,02 | 10 | 0,9 |
| Piston | LC411 | 397,8 | 122 | 4,77 | 25 | 28 | 129 | 65107 | 16556 | 0,02 | 32 | 1,1 |
| Piston | LC409 | 398,8 | 115 | 4,89 | 14 | 28 | 138 | 67579 | 11639 | 0,01 | 15 | 0,6 |
| Piston | LC407 | 399,8 | 108 | 4,72 | 6 | 27 | 131 | 68479 | 12255 | 0,06 | 4 | 0,3 |
| Piston | LC406 | 400,2 | 105 | 4,81 | 8 | 26 | 131 | 69474 | 10461 | 0,05 | 4 | 0,5 |
| Piston | LC405 | 400,7 | 102 | 4,80 | 15 | 27 | 136 | 68912 | 15642 | 0,03 | 5 | 0,7 |
| Piston | LC403 | 401,7 | 96 | 4,86 | 12 | 30 | 144 | 70952 | 11521 | 0,01 | 3 | 0,7 |
| Piston | LC401 | 402,7 | 89 | 4,69 | 10 | 26 | 127 | 67832 | 13747 | 0 | 30 | 0,5 |
| Piston | LC397 | 404,7 | 76 | 4,91 | 9 | 26 | 138 | 69655 | 12433 | 0,02 | 9 | 0,4 |
| Piston | LC393 | 406,7 | 63 | 5,23 | 17 | 26 | 137 | 65993 | 15214 | 0,01 | 16 | 0,8 |
| Piston | LC389 | 408,6 | 50 | 4,80 | 16 | 26 | 130 | 63009 | 16164 | 0 | 55 | 0,8 |
| Piston | LC385 | 410,6 | 37 | 5,39 | 25 | 27 | 133 | 69188 | 11022 | 0,05 | 5 | 1,3 |
| Piston | LC382 | 412,1 | 28 | 5,14 | 11 | 27 | 134 | 66135 | 16721 | 0 | 26 | 0,6 |
| Piston | LC379 | 413,5 | 19 | 4,95 | 16 | 25 | 132 | 66779 | 14607 | 0 | 16 | 0,9 |
| Piston | LC376 | 415,0 | 10 | 5,05 | 12 | 26 | 133 | 69138 | 8885 | 0,01 | 4 | 0,7 |
| Piston | LC372 | 417,0 | -2 | 5,13 | 16 | 27 | 137 | 67775 | 11794 | 0,01 | 5 | 0,9 |
| Piston | LC368 | 419,0 | -14 | 5,30 | 19 | 26 | 131 | 69670 | 12502 | 0,03 | 4 | 1,1 |
| Piston | LC364 | 420,9 | -24 | 5,15 | 14 | 28 | 140 | 67734 | 17622 | 0,01 | 6 | 0,9 |
| Piston | LC360 | 422,9 | -36 | 5,36 | 23 | 27 | 141 | 67950 | 14120 | 0,05 | 4 | 1,3 |
| Piston | LC355 | 425,4 | -50 | 5,78 | 21 | 28 | 149 | 67978 | 13831 | 0,01 | 4 | 1,2 |
| Piston | LC351 | 427,3 | -58 | 5,37 | 21 | 26 | 136 | 69558 | 18315 | 0,03 | 4 | 1,7 |
| Piston | LC347 | 429,3 | -66 | 5,02 | 30 | 26 | 139 | 67099 | 19444 | 0 | 18 | 2,4 |
| Piston | LC343 | 431,3 | -75 | 5,14 | 15 | 29 | 140 | 68223 | 13115 | 0 | 7 | 1,0 |
| Piston | LC339 | 433,3 | -83 | 5,02 | 11 | 26 | 139 | 68628 | 9601 | 0,01 | 19 | 0,9 |
| Piston | LC335 | 435,2 | -91 | 5,16 | 21 | 26 | 133 | 69218 | 16367 | 0,02 | 7 | 1,8 |
| Piston | LC330 | 437,7 | -100 | 5,38 | 9 | 27 | 148 | 69084 | 8202 | 0,01 | 3 | 0,8 |
| Piston | LC326 | 439,7 | -107 | 5,01 | 10 | 25 | 134 | 66905 | 16920 | 0,01 | 17 | 1,0 |
| Piston | LC322 | 441,6 | -112 | 5,03 | 13 | 28 | 151 | 68841 | 12772 | 0,01 | 4 | 1,4 |
| Piston | LC318 | 443,6 | -120 | 5,21 | 20 | 24 | 132 | 69859 | 11889 | 0,03 | 6 | 1,6 |
| Piston | LC314 | 445,6 | -128 | 5,29 | 20 | 25 | 137 | 67610 | 12028 | 0,01 | 14 | 1,6 |
| Piston | LC310 | 447,5 | -136 | 5,58 | 19 | 27 | 142 | 68976 | 10351 | 0,02 | 3 | 1,6 |
| Piston | LC306 | 449,5 | -145 | 5,01 | 34 | 23 | 132 | 64814 | 16560 | 0 | 40 | 2,3 |
| Piston | LC302 | 451,5 | -153 | 5,04 | 26 | 24 | 134 | 68320 | 18893 | 0 | 5 | 2,1 |
| Piston | LC298 | 453,4 | -165 | 5,10 | 22 | 25 | 143 | 67476 | 12952 | 0,01 | 5 | 1,2 |
| Piston | LC294 | 455,4 | -181 | 4,75 | 22 | 26 | 141 | 68220 | 14153 | 0,01 | 4 | 0,9 |
| Piston | LC290 | 457,4 | -198 | 4,57 | 8 | 25 | 136 | 68616 | 11327 | 0,02 | 6 | 0,3 |
| Piston | LC286 | 459,4 | -214 | 4,62 | 14 | 25 | 131 | 71928 | 12532 | 0,03 | 5 | 0,6 |
| Piston | LC282 | 461,3 | -229 | 4,72 | 14 | 26 | 140 | 68591 | 11130 | 0,01 | 5 | 0,6 |
| Piston | LC278 | 463,3 | -245 | 4,81 | 9 | 25 | 136 | 67730 | 10914 | 0,02 | 14 | 0,4 |
| Piston | LC273 | 465,8 | -266 | 5,14 | 18 | 27 | 140 | 67940 | 16873 | 0,02 | 5 | 0,8 |
| Piston | LC269 | 467,7 | -282 | 5,06 | 16 | 26 | 132 | 69493 | 9065 | 0,04 | 13 | 0,7 |
| Piston | LC266 | 469,2 | -294 | 4,88 | 13 | 26 | 139 | 70424 | 10172 | 0,02 | 4 | 0,5 |
| Piston | LC262 | 471,2 | -310 | 5,12 | 18 | 25 | 142 | 67798 | 16195 | 0,02 | 5 | 0,7 |
| Piston | LC258 | 473,2 | -325 | 5,06 | 26 | 25 | 142 | 65288 | 22516 | 0,01 | 16 | 1,2 |
| Piston | LC254 | 475,1 | -339 | 4,84 | 12 | 25 | 139 | 69430 | 11669 | 0,01 | 4 | 0,5 |
| Piston | LC250 | 477,1 | -353 | 4,87 | 14 | 24 | 134 | 67361 | 17346 | 0 | 13 | 0,6 |
| Piston | LC246 | 479,1 | -368 | 4,90 | 17 | 24 | 135 | 64562 | 11608 | 0,01 | 28 | 0,7 |
| Piston | LC242 | 481,0 | -383 | 4,80 | 13 | 25 | 136 | 67891 | 11288 | 0 | 7 | 0,6 |
| Piston | LC238 | 483,0 | -398 | 4,91 | 11 | 26 | 138 | 69426 | 9278 | 0,01 | 6 | 0,5 |
| Piston | LC234 | 485,0 | -413 | 4,77 | 16 | 24 | 130 | 67334 | 12832 | 0,03 | 23 | 0,7 |
| Piston | LC230 | 486,9 | -427 | 4,93 | 13 | 25 | 136 | 68274 | 11913 | 0,01 | 4 | 0,6 |
| Piston | LC226 | 488,9 | -442 | 4,79 | 9 | 21 | 135 | 67415 | 15489 | 0,02 | 9 | 0,4 |
| Piston | LC221 | 491,4 | -461 | 4,91 | 20 | 25 | 134 | 70740 | 12440 | 0,03 | 8 | 0,8 |
| Piston | LC216 | 493,8 | -479 | 4,84 | 10 | 24 | 134 | 68315 | 12738 | 0 | 9 | 0,4 |
| Piston | LC211 | 496,3 | -498 | 4,94 | 13 | 25 | 137 | 67067 | 10436 | 0 | 16 | 0,5 |
| Piston | LC206 | 498,8 | -511 | 5,22 | 11 | 26 | 137 | 66990 | 9564 | 0,04 | 10 | 0,7 |
| Piston | LC202 | 500,7 | -521 | 5,00 | 21 | 25 | 136 | 69219 | 13579 | 0,03 | 9 | 1,2 |
| Piston | LC198 | 502,7 | -532 | 5,29 | 15 | 25 | 138 | 67610 | 12625 | 0,01 | 10 | 0,9 |
| Piston | LC194 | 504,7 | -543 | 4,92 | 12 | 26 | 138 | 68387 | 11384 | 0 | 6 | 0,7 |
| Piston | LC190 | 506,7 | -554 | 4,98 | 11 | 24 | 137 | 66732 | 11771 | 0 | 9 | 0,6 |
| Piston | LC186 | 508,6 | -565 | 5,01 | 12 | 23 | 129 | 67701 | 12383 | 0,04 | 12 | 0,7 |
| Piston | LC182 | 510,6 | -577 | 5,28 | 13 | 27 | 140 | 66160 | 11820 | 0,01 | 15 | 0,7 |
| Piston | LC178 | 512,6 | -588 | 5,40 | 16 | 23 | 130 | 59730 | 14196 | 0 | 69 | 0,9 |
| Piston | LC174 | 514,5 | -598 | 5,50 | 13 | 23 | 138 | 64495 | 13085 | 0,02 | 33 | 0,8 |
| Piston | LC169 | 517,0 | -612 | 5,51 | 27 | 24 | 186 | 66401 | 14038 | 0,02 | 29 | 1,5 |
| Piston | LC165 | 519,0 | -624 | 5,10 | 22 | 37 | 127 | 58873 | 36637 | 0 | 108 | 1,2 |
| Piston | LC160 | 521,4 | -637 | 5,09 | 16 | 25 | 135 | 66245 | 9997 | 0,01 | 13 | 1,0 |
| Piston | LC156 | 523,4 | -648 | 5,02 | 19 | 24 | 131 | 63422 | 14523 | 0 | 36 | 1,1 |
| Piston | LC152 | 525,4 | -659 | 5,02 | 24 | 21 | 125 | 62786 | 12839 | 0 | 38 | 1,4 |
| Piston | LC148 | 527,3 | -670 | 5,23 | 16 | 26 | 132 | 67488 | 14636 | 0 | 7 | 0,9 |
| Piston | LC144 | 529,3 | -682 | 5,33 | 15 | 25 | 132 | 64891 | 12751 | 0 | 10 | 0,8 |
| Piston | LC140 | 531,3 | -695 | 4,55 | 11 | 25 | 133 | 66087 | 10855 | 0,01 | 4 | 0,6 |
| Piston | LC136 | 533,3 | -707 | 5,26 | 13 | 23 | 134 | 68953 | 13683 | 0,04 | 5 | 0,7 |
| Piston | LC132 | 535,2 | -719 | 4,74 | 15 | 25 | 135 | 68802 | 8298 | 0,02 | 3 | 0,8 |
| Piston | LC128 | 537,2 | -731 | 5,22 | 17 | 27 | 133 | 65590 | 14642 | 0 | 23 | 0,9 |
| Piston | LC124 | 539,2 | -743 | 5,41 | 15 | 28 | 136 | 66386 | 13571 | 0,01 | 6 | 0,8 |
| Piston | LC119 | 541,6 | -757 | 5,20 | 15 | 23 | 126 | 69084 | 10486 | 0,03 | 4 | 0,9 |
| Piston | LC115 | 543,6 | -769 | 5,34 | 12 | 24 | 134 | 66800 | 12205 | 0,01 | 5 | 0,6 |
| Piston | LC110 | 546,1 | -783 | 5,63 | 13 | 24 | 137 | 66426 | 14485 | 0,01 | 4 | 0,8 |
| Piston | LC106 | 548,0 | -808 | 5,46 | 13 | 23 | 136 | 66543 | 12036 | 0 | 5 | 0,3 |
| Piston | LC102 | 550,0 | -823 | 5,47 | 15 | 25 | 130 | 68501 | 13007 | 0,03 | 5 | 0,7 |
| Piston | LC98 | 552,0 | -835 | 5,43 | 8 | 21 | 131 | 66117 | 13367 | 0,01 | 22 | 0,5 |
| Piston | LC94 | 553,9 | -845 | 5,52 | 10 | 25 | 132 | 66359 | 15216 | 0,01 | 8 | 0,6 |
| Piston | LC89 | 556,4 | -859 | 5,29 | 7 | 24 | 132 | 67625 | 13530 | 0,02 | 5 | 0,4 |
| Piston | LC85 | 558,4 | -870 | 5,15 | 11 | 26 | 127 | 67524 | 13374 | 0,05 | 12 | 0,6 |
| Piston | LC80 | 560,8 | -882 | 5,42 | 16 | 26 | 139 | 68059 | 12680 | 0,01 | 6 | 1,0 |
| Piston | LC75 | 563,3 | -894 | 5,33 | 11 | 23 | 135 | 66454 | 15533 | 0,01 | 19 | 0,7 |
| Piston | LC71 | 565,3 | -904 | 5,55 | 13 | 26 | 142 | 68914 | 16123 | 0,02 | 9 | 0,8 |
| Piston | LC67 | 567,2 | -914 | 5,45 | 21 | 22 | 129 | 65589 | 14089 | 0,03 | 25 | 1,5 |
| Piston | LC64 | 568,7 | -921 | 5,72 | 26 | 24 | 141 | 68026 | 14359 | 0,02 | 5 | 1,7 |
| Piston | LC60 | 570,7 | -931 | 5,65 | 17 | 26 | 142 | 67420 | 13623 | 0,02 | 10 | 1,1 |
| Piston | LC56 | 572,7 | -941 | 5,77 | 21 | 24 | 142 | 67418 | 17767 | 0,01 | 5 | 1,3 |
| Piston | LC52 | 574,6 | -952 | 5,71 | 21 | 25 | 136 | 67947 | 17180 | 0,03 | 5 | 1,3 |
| Piston | LC36 | 582,5 |  | 5,55 | 18 | 25 | 133 | 68805 | 19702 | 0,03 | 5 | 1,4 |
| Piston | LC19 | 590,9 |  | 5,39 | 13 | 24 | 133 | 69265 | 14551 | 0,03 | 8 | 1,5 |
| Piston | LC3 | 598,8 |  | 5,59 | 13 | 25 | 140 | 70097 | 14749 | 0,04 | 7 | 1,5 |

**Table S2.** Median calendar age, TEX_86_^L^ (Kim et al. 2010), BIT (Hopmans et al. 2004) and TEX_86_^L^-based sea surface temperature (SST) reconstructions, using the Baltic Sea calibration by Kabel et al. (2012) for Erstaviken.

| **Core** | **Labcode** | **Composite depth (cmbsf)** | | **Median calendar age** | **TEX_86_^L^** | **BIT** | **SST (Kabel et al., 2012)** |
| --- | --- | --- | --- | --- | --- | --- | --- |
| Gemini | MC2 | | 1,5 | 2013 | -0,63 | 0,05 | 15,2 |
| Gemini | MC15 | | 14,5 | 2000 | -0,66 | 0,07 | 14,3 |
| Piston | LC1226 | | 29,1 | 1992 | -0,67 | 0,07 | 13,9 |
| Piston | LC1154 | | 48,6 | 1958 | -0,69 | 0,07 | 13,3 |
| Piston | LC1122 | | 56,8 | 1958 | -0,70 | 0,08 | 12,8 |
| Piston | LC1078 | | 71,9 | 1932 | -0,68 | 0,09 | 13,6 |
| Piston | LC1060 | | 80,0 | 1896 | -0,67 | 0,09 | 13,9 |
| Piston | LC1043 | | 87,6 | 1863 | -0,72 | 0,09 | 12,3 |
| Piston | LC1022 | | 97,1 | 1821 | -0,73 | 0,10 | 12,0 |
| Piston | LC1005 | | 105,2 | 1787 | -0,76 | 0,10 | 11,0 |
| Piston | LC988 | | 113,5 | 1753 | -0,75 | 0,10 | 11,4 |
| Piston | LC973 | | 120,9 | 1723 | -0,73 | 0,11 | 12,1 |
| Piston | LC953 | | 130,8 | 1683 | -0,72 | 0,10 | 12,3 |
| Piston | LC941 | | 136,7 | 1659 | -0,73 | 0,11 | 11,9 |
| Piston | LC930 | | 142,1 | 1638 | -0,73 | 0,12 | 12,0 |
| Piston | LC914 | | 150,0 | 1606 | -0,71 | 0,10 | 12,7 |
| Piston | LC900 | | 156,9 | 1578 | -0,72 | 0,12 | 12,1 |
| Piston | LC886 | | 163,8 | 1551 | -0,74 | 0,10 | 11,5 |
| Piston | LC881 | | 166,3 | 1542 | -0,73 | 0,11 | 12,0 |
| Piston | LC872 | | 170,7 | 1524 | -0,73 | 0,11 | 11,9 |
| Piston | LC831 | | 190,9 | 1446 | -0,70 | 0,09 | 12,7 |
| Piston | LC830 | | 191,4 | 1444 | -0,72 | 0,09 | 12,3 |
| Piston | LC817 | | 197,8 | 1421 | -0,71 | 0,09 | 12,4 |
| Piston | LC812 | | 200,3 | 1412 | -0,71 | 0,09 | 12,6 |
| Piston | LC795 | | 208,8 | 1380 | -0,71 | 0,08 | 12,5 |
| Piston | LC774 | | 219,3 | 1340 | -0,69 | 0,08 | 13,2 |
| Piston | LC767 | | 222,8 | 1327 | -0,71 | 0,10 | 12,5 |
| Piston | LC753 | | 229,8 | 1301 | -0,70 | 0,10 | 12,8 |
| Piston | LC746 | | 233,3 | 1287 | -0,71 | 0,09 | 12,5 |
| Piston | LC728 | | 242,3 | 1253 | -0,68 | 0,08 | 13,4 |
| Piston | LC720 | | 246,3 | 1237 | -0,70 | 0,09 | 13,0 |
| Piston | LC702 | | 255,3 | 1202 | -0,70 | 0,08 | 12,9 |
| Piston | LC694 | | 259,3 | 1186 | -0,69 | 0,08 | 13,2 |
| Piston | LC667 | | 272,8 | 1130 | -0,68 | 0,09 | 13,5 |
| Piston | LC646 | | 283,3 | 1086 | -0,69 | 0,09 | 13,4 |
| Piston | LC635 | | 288,8 | 1064 | -0,68 | 0,08 | 13,5 |
| Piston | LC621 | | 295,8 | 1036 | -0,63 | 0,11 | 15,3 |
| Piston | LC600 | | 306,1 | 947 | -0,68 | 0,08 | 13,5 |
| Piston | LC593 | | 309,5 | 911 | -0,68 | 0,08 | 13,4 |
| Piston | LC581 | | 315,3 | 848 | -0,71 | 0,08 | 12,7 |
| Piston | LC576 | | 317,7 | 823 | -0,69 | 0,07 | 13,3 |
| Piston | LC567 | | 322,1 | 775 | -0,70 | 0,09 | 12,8 |
| Piston | LC555 | | 327,9 | 705 | -0,68 | 0,09 | 13,7 |
| Piston | LC547 | | 331,8 | 670 | -0,69 | 0,08 | 13,2 |
| Piston | LC531 | | 339,6 | 607 | -0,69 | 0,07 | 13,3 |
| Piston | LC527 | | 341,5 | 592 | -0,70 | 0,08 | 12,8 |
| Piston | LC515 | | 347,3 | 546 | -0,70 | 0,07 | 12,8 |
| Piston | LC512 | | 348,8 | 534 | -0,70 | 0,08 | 12,7 |
| Piston | LC503 | | 351,7 | 509 | -0,72 | 0,08 | 12,2 |
| Piston | LC496 | | 356,6 | 470 | -0,69 | 0,07 | 13,3 |
| Piston | LC484 | | 362,4 | 422 | -0,71 | 0,07 | 12,5 |
| Piston | LC479 | | 364,8 | 402 | -0,70 | 0,07 | 13,0 |
| Piston | LC466 | | 371,1 | 348 | -0,72 | 0,08 | 12,4 |
| Piston | LC450 | | 378,9 | 279 | -0,70 | 0,08 | 12,9 |
| Piston | LC446 | | 380,8 | 262 | -0,71 | 0,09 | 12,6 |
| Piston | LC425 | | 391,0 | 175 | -0,66 | 0,10 | 14,1 |
| Piston | LC406 | | 400,2 | 105 | -0,70 | 0,08 | 12,9 |
| Piston | LC379 | | 413,5 | 19 | -0,73 | 0,08 | 12,0 |
| Piston | LC339 | | 433,3 | -83 | -0,69 | 0,12 | 13,2 |
| Piston | LC302 | | 451,5 | -153 | -0,70 | 0,06 | 13,0 |
| Piston | LC278 | | 463,3 | -245 | -0,70 | 0,07 | 12,8 |
| Piston | LC258 | | 473,2 | -325 | -0,72 | 0,07 | 12,1 |
| Piston | LC230 | | 486,9 | -427 | -0,73 | 0,07 | 12,0 |
| Piston | LC202 | | 500,7 | -521 | -0,72 | 0,07 | 12,1 |
| Piston | LC182 | | 510,6 | -577 | -0,70 | 0,07 | 12,7 |
| Piston | LC156 | | 523,4 | -648 | -0,69 | 0,07 | 13,2 |
| Piston | LC128 | | 537,2 | -731 | -0,72 | 0,07 | 12,4 |
| Piston | LC102 | | 550,0 | -823 | -0,71 | 0,06 | 12,7 |
| Piston | LC67 | | 567,2 | -914 | -0,71 | 0,06 | 12,5 |

**Table S3.** Sedimentary concentrations of organic carbon (C_org_), molybdenum (Mo), lead (Pb), and zinc (Zn) for Baggensfjärden.

| **Core** | **Labcode** | **Composite depth**  **(cmbsf)** | **C_org_**  **(wt.%)** | **Mo**  **(ppm)** | **Pb**  **(ppm)** | **Zn**  **(ppm)** |
| --- | --- | --- | --- | --- | --- | --- |
| Gemini | MC1 | 0,5 | 7,02 | 13 | 57 | 279 |
| Gemini | MC2 | 1,5 | 5,62 | 17 | 64 | 307 |
| Gemini | MC3 | 2,5 | 4,75 | 7 | 79 | 267 |
| Gemini | MC4 | 3,5 | 4,65 | 8 | 77 | 265 |
| Gemini | MC5 | 4,5 | 5,05 | 9 | 80 | 299 |
| Gemini | MC6 | 5,5 | 4,92 | 12 | 79 | 313 |
| Gemini | MC7 | 6,5 | 4,74 | 10 | 83 | 296 |
| Gemini | MC8 | 7,5 | 4,82 | 10 | 88 | 305 |
| Gemini | MC9 | 8,5 | 4,81 | 11 | 96 | 329 |
| Gemini | MC10 | 9,5 | 4,65 | 9 | 122 | 335 |
| Gemini | MC11 | 10,5 | 4,54 | 6 | 133 | 332 |
| Gemini | MC12 | 11,5 | 4,56 | 7 | 137 | 343 |
| Gemini | MC13 | 12,5 | 4,63 | 12 | 175 | 427 |
| Gemini | MC14 | 13,5 | 4,75 | 12 | 190 | 427 |
| Gemini | MC15 | 14,5 | 4,96 | 15 | 186 | 467 |
| Gemini | MC16 | 15,5 | 4,50 | 12 | 138 | 396 |
| Gemini | MC17 | 16,5 | 4,43 | 10 | 116 | 372 |
| Gemini | MC18 | 17,5 | 4,59 | 10 | 121 | 421 |
| Gemini | MC19 | 18,5 | 4,67 | 10 | 117 | 413 |
| Gemini | MC20 | 19,5 | 4,51 | 7 | 112 | 327 |
| Gemini | MC21 | 20,5 | 4,39 | 5 | 109 | 300 |
| Gemini | MC22 | 21,5 | 4,35 | 4 | 104 | 295 |
| Gemini | MC23 | 22,5 | 4,31 | 4 | 107 | 300 |
| Gemini | MC24 | 23,5 | 4,33 | 4 | 99 | 301 |
| Gemini | MC25 | 24,5 | 4,18 | 4 | 101 | 295 |
| Gemini | MC26 | 25,5 | 4,16 | 3 | 96 | 270 |
| Gemini | MC27 | 26,5 | 4,36 | 4 | 106 | 277 |
| Gemini | MC28 | 27,5 | 4,34 | 5 | 99 | 274 |
| Gemini | MC29 | 28,5 | 4,43 | 7 | 101 | 295 |
| Gemini | MC30 | 29,5 | 4,53 | 7 | 101 | 307 |
| Gemini | MC31 | 30,5 | 4,40 | 9 | 99 | 313 |
| Gemini | MC32 | 31,5 | 4,28 | 3 | 100 | 285 |
| Gemini | MC33 | 32,5 | 4,33 | 1 | 104 | 285 |
| Gemini | MC34 | 33,5 | 4,39 | 1 | 119 | 274 |
| Gemini | MC35 | 34,5 | 4,59 | 1 | 126 | 270 |
| Gemini | MC36 | 35,5 | 4,24 | 2 | 125 | 265 |
| Gemini | MC37 | 36,5 | 4,26 | 1 | 131 | 255 |
| Gemini | MC38 | 37,5 | 4,37 | 1 | 133 | 263 |
| Gemini | MC39 | 38,5 | 4,42 | 3 | 134 | 280 |
| Gemini | MC40 | 39,5 | 4,41 | 1 | 132 | 278 |
| Gemini | MC41 | 40,5 | 4,40 | 1 | 128 | 271 |
| Gemini | MC42 | 41,5 | 4,27 | 1 | 122 | 256 |
| Gemini | MC43 | 42,5 | 4,36 | 1 | 106 | 240 |
| Gemini | MC44 | 43,5 | 4,43 | 1 | 102 | 241 |
| Gemini | MC45 | 44,5 | 4,39 | 1 | 101 | 240 |
| Gemini | MC46 | 45,5 | 4,36 | 2 | 94 | 235 |
| Gemini | MC47 | 46,5 | 4,34 | 2 | 90 | 235 |
| Gemini | MC48 | 47,5 | 4,19 | 5 | 89 | 234 |
| Gemini | MC49 | 48,5 | 4,17 | 1 | 92 | 224 |
| Gemini | MC50 | 49,5 | 4,20 | 0 | 89 | 223 |
| Gemini | MC51 | 50,5 | 4,23 | 2 | 89 | 229 |
| Gemini | MC52 | 51,5 | 4,13 | 1 | 90 | 228 |
| Gemini | MC53 | 52,5 | 4,14 | 2 | 90 | 223 |
| Gemini | MC54 | 53,5 | 4,10 | 1 | 89 | 224 |
| Piston | LC59 | 54,5 | 4,22 | 8 | 91 | 294 |
| Gemini | MC55 | 54,5 | 4,19 | 2 | 89 | 227 |
| Gemini | MC56 | 55,5 | 4,20 | 8 | 88 | 236 |
| Gemini | MC57 | 56,5 | 4,30 | 6 | 92 | 245 |
| Gemini | MC58 | 57,5 | 4,30 | 2 | 91 | 240 |
| Gemini | MC59 | 58,5 | 4,29 | 1 | 92 | 243 |
| Piston | LC58 | 59,5 | 4,32 | 5 | 94 | 268 |
| Gemini | MC60 | 59,5 | 4,33 | 2 | 90 | 234 |
| Piston | LC57 | 64,5 | 3,41 | 12 | 67 | 230 |
| Piston | LC56 | 69,5 | 3,56 | 10 | 32 | 162 |
| Piston | LC55 | 74,5 | 3,70 | 13 | 33 | 199 |
| Piston | LC54 | 79,5 | 4,29 | 13 | 33 | 153 |
| Piston | LC53 | 86 | 3,98 | 24 | 28 | 145 |
| Piston | LC52 | 93,5 | 4,67 | 14 | 31 | 147 |
| Piston | LC51 | 101,5 | 4,88 | 26 | 27 | 160 |
| Piston | LC50 | 111,3 | 4,81 | 32 | 25 | 156 |
| Piston | LC49 | 121,1 | 4,11 | 36 | 23 | 164 |
| Piston | LC48 | 130,9 | 5,09 | 37 | 21 | 154 |
| Piston | LC47 | 140,7 | 4,58 | 25 | 23 | 146 |
| Piston | LC46 | 150,5 | 4,88 | 43 | 22 | 143 |
| Piston | LC45 | 160,3 | 5,24 | 43 | 21 | 156 |
| Piston | LC44 | 170,1 | 4,88 | 33 | 22 | 151 |
| Piston | LC43 | 179,9 | 5,16 | 29 | 21 | 147 |
| Piston | LC42 | 189,7 | 5,37 | 37 | 19 | 144 |
| Piston | LC41 | 199,5 | 5,68 | 31 | 21 | 138 |
| Piston | LC40 | 209,5 | 5,83 | 21 | 21 | 158 |
| Piston | LC39 | 219,5 | 5,61 | 21 | 20 | 132 |
| Piston | LC38 | 229,5 | 5,45 | 18 | 22 | 138 |
| Piston | LC37 | 239,5 | 5,53 | 19 | 21 | 136 |
| Piston | LC36 | 249,5 | 5,13 | 18 | 21 | 142 |
| Piston | LC35 | 259,5 | 4,90 | 12 | 20 | 133 |
| Piston | LC34 | 269,5 | 5,22 | 14 | 22 | 141 |
| Piston | LC33 | 279,5 | 5,15 | 17 | 22 | 138 |
| Piston | LC32 | 289,5 | 6,09 | 12 | 22 | 133 |
| Piston | LC31 | 299,5 | 5,66 | 10 | 22 | 135 |
| Piston | LC30 | 309,5 | 5,93 | 7 | 21 | 129 |
| Piston | LC29 | 319,5 | 4,43 | 8 | 19 | 128 |
| Piston | LC28 | 329,5 | 5,86 | 6 | 21 | 128 |
| Piston | LC27 | 339,5 | 5,74 | 6 | 22 | 132 |
| Piston | LC26 | 349,5 | 5,78 | 8 | 22 | 129 |
| Piston | LC25 | 359,5 | 5,57 | 5 | 21 | 130 |
| Piston | LC24 | 369,5 | 5,62 | 8 | 22 | 133 |
| Piston | LC23 | 379,5 | 5,32 | 7 | 21 | 127 |
| Piston | LC22 | 389,5 | 4,69 | 4 | 19 | 119 |
| Piston | LC21 | 399,5 | 1,40 | 4 | 23 | 128 |
| Piston | LC20 | 409,5 | 1,69 | 3 | 23 | 129 |
| Piston | LC19 | 419,5 | 1,97 | 2 | 23 | 130 |
| Piston | LC18 | 429,5 | 1,75 | 3 | 23 | 131 |
| Piston | LC17 | 439,5 | 1,55 | 3 | 21 | 131 |
| Piston | LC16 | 449,5 | 1,54 | 4 | 24 | 135 |
| Piston | LC15 | 459,5 | 1,41 | 4 | 25 | 141 |
| Piston | LC14 | 469,5 | 1,09 | 3 | 25 | 142 |
| Piston | LC13 | 479,5 | 1,24 | 4 | 23 | 136 |
| Piston | LC12 | 489,5 | 1,13 | 2 | 25 | 136 |
| Piston | LC11 | 499,5 | 1,08 | 2 | 25 | 135 |
| Piston | LC10 | 509,5 | 0,68 | 2 | 25 | 138 |
| Piston | LC9 | 519,5 | 0,62 | 2 | 21 | 139 |
| Piston | LC8 | 529,5 | 0,67 | 0 | 25 | 132 |
| Piston | LC7 | 539,5 | 0,70 | 1 | 27 | 140 |
| Piston | LC6 | 549,5 | 0,61 | 2 | 24 | 125 |
| Piston | LC5 | 559,5 | 0,64 | 2 | 21 | 106 |
| Piston | LC4 | 569,5 | 0,65 | 1 | 19 | 99 |
| Piston | LC3 | 579,5 | 0,98 | 1 | 26 | 134 |
| Piston | LC2 | 589,5 | 1,01 | 2 | 25 | 126 |
| Piston | LC1 | 599,5 | 1,58 | 3 | 21 | 123 |

**Table S4.** Sedimentary concentrations of organic carbon (C_org_), molybdenum (Mo), lead (Pb), and zinc (Zn) for Ingaröfjärden.

| **Core** | **Labcode** | **Composite**  **depth**  **(cmbsf)** | **C_org_**  **(wt.%)** | **Mo**  **(ppm)** | **Pb**  **(ppm)** | **Zn**  **(ppm)** |
| --- | --- | --- | --- | --- | --- | --- |
| Gemini | MC1 | 0,5 | 5,17 | 3 | 38 | 171 |
| Gemini | MC2 | 1,5 | 5,13 | 3 | 39 | 178 |
| Gemini | MC3 | 2,5 | 4,85 | 2 | 37 | 167 |
| Gemini | MC4 | 3,5 | 4,83 | 3 | 41 | 183 |
| Gemini | MC5 | 4,5 | 4,39 | 3 | 39 | 179 |
| Gemini | MC6 | 5,5 | 4,20 | 6 | 40 | 180 |
| Gemini | MC7 | 6,5 | 4,17 | 7 | 49 | 185 |
| Gemini | MC8 | 7,5 | 4,46 | 4 | 51 | 202 |
| Gemini | MC9 | 8,5 | 4,62 | 4 | 59 | 216 |
| Gemini | MC10 | 9,5 | 4,69 | 4 | 54 | 216 |
| Piston | LC59 | 10,5 | 4,84 | 1 | 58 | 243 |
| Gemini | MC11 | 10,5 | 4,65 | 4 | 58 | 228 |
| Gemini | MC12 | 11,5 | 4,47 | 3 | 57 | 230 |
| Gemini | MC13 | 12,5 | 4,39 | 3 | 55 | 221 |
| Gemini | MC14 | 13,5 | 4,15 | 4 | 52 | 211 |
| Gemini | MC15 | 14,5 | 3,78 | 5 | 53 | 198 |
| Piston | LC58 | 15,5 | 4,33 | 3 | 51 | 208 |
| Gemini | MC16 | 15,5 | 4,01 | 6 | 52 | 211 |
| Gemini | MC17 | 16,5 | 4,03 | 5 | 52 | 211 |
| Gemini | MC18 | 17,5 | 3,97 | 7 | 53 | 215 |
| Gemini | MC19 | 18,5 | 3,94 | 8 | 53 | 207 |
| Gemini | MC20 | 19,5 | 4,24 | 6 | 57 | 216 |
| Gemini | MC21 | 20,5 | 3,94 | 6 | 55 | 207 |
| Gemini | MC22 | 21,5 | 3,75 | 6 | 52 | 197 |
| Piston | LC57 | 22,5 | 3,83 | 3 | 49 | 181 |
| Gemini | MC23 | 22,5 | 3,72 | 4 | 52 | 204 |
| Gemini | MC24 | 23,5 | 3,57 | 6 | 55 | 193 |
| Gemini | MC25 | 24,5 | 3,59 | 6 | 56 | 190 |
| Gemini | MC26 | 25,5 | 3,61 | 8 | 56 | 194 |
| Gemini | MC27 | 26,5 | 3,77 | 6 | 57 | 193 |
| Gemini | MC28 | 27,5 | 3,51 | 6 | 44 | 166 |
| Gemini | MC29 | 28,5 | 3,40 | 6 | 45 | 157 |
| Gemini | MC30 | 29,5 | 3,39 | 4 | 43 | 152 |
| Gemini | MC31 | 30,5 | 3,21 | 7 | 36 | 147 |
| Piston | LC56 | 31,5 | 3,61 | 1 | 39 | 149 |
| Gemini | MC32 | 31,5 | 3,43 | 9 | 41 | 158 |
| Gemini | MC33 | 32,5 | 3,51 | 5 | 41 | 161 |
| Gemini | MC34 | 33,5 | 3,56 | 4 | 38 | 163 |
| Gemini | MC35 | 34,5 | 3,62 | 5 | 38 | 158 |
| Gemini | MC36 | 35,5 | 3,65 | 4 | 41 | 163 |
| Gemini | MC37 | 36,5 | 3,60 | 4 | 38 | 164 |
| Gemini | MC38 | 37,5 | 3,57 | 5 | 38 | 153 |
| Gemini | MC39 | 38,5 | 3,61 | 5 | 39 | 163 |
| Gemini | MC40 | 39,5 | 3,60 | 4 | 39 | 156 |
| Gemini | MC41 | 40,5 | 3,64 | 3 | 36 | 154 |
| Gemini | MC42 | 41,5 | 3,73 | 3 | 33 | 150 |
| Piston | LC55 | 42,5 | 3,76 | 2 | 34 | 147 |
| Gemini | MC43 | 42,5 | 3,68 | 4 | 37 | 145 |
| Gemini | MC44 | 43,5 | 3,65 | 6 | 34 | 151 |
| Gemini | MC45 | 44,5 | 3,73 | 5 | 34 | 150 |
| Gemini | MC46 | 45,5 | 3,61 | 6 | 34 | 147 |
| Gemini | MC47 | 46,5 | 3,65 | 7 | 38 | 142 |
| Gemini | MC48 | 47,5 | 3,62 | 7 | 37 | 141 |
| Gemini | MC49 | 48,5 | 3,76 | 6 | 35 | 141 |
| Gemini | MC50 | 49,5 | 3,94 | 6 | 34 | 155 |
| Gemini | MC51 | 50,5 | 4,03 | 5 | 35 | 147 |
| Gemini | MC52 | 51,5 | 4,10 | 4 | 37 | 147 |
| Gemini | MC53 | 52,5 | 4,06 | 8 | 34 | 151 |
| Gemini | MC54 | 53,5 | 4,00 | 6 | 35 | 154 |
| Gemini | MC55 | 54,5 | 4,03 | 5 | 34 | 145 |
| Piston | LC54 | 55,5 | 3,91 | 7 | 32 | 134 |
| Gemini | MC56 | 55,5 | 4,10 | 5 | 35 | 142 |
| Gemini | MC57 | 56,5 | 4,08 | 7 | 34 | 142 |
| Gemini | MC58 | 57,5 | 4,14 | 6 | 35 | 149 |
| Gemini | MC59 | 58,5 | 4,26 | 7 | 36 | 150 |
| Gemini | MC60 | 59,5 | 4,19 | 6 | 33 | 142 |
| Piston | LC53 | 70,5 | 4,26 | 6 | 32 | 129 |
| Piston | LC52 | 85,5 | 3,78 | 10 | 29 | 127 |
| Piston | LC51 | 95,5 | 4,40 | 7 | 31 | 136 |
| Piston | LC50 | 105,5 | 4,86 | 10 | 29 | 136 |
| Piston | LC49 | 115,5 | 4,91 | 10 | 27 | 137 |
| Piston | LC48 | 125,5 | 5,17 | 14 | 25 | 131 |
| Piston | LC47 | 135,5 | 4,89 | 14 | 23 | 132 |
| Piston | LC46 | 145,5 | 5,09 | 10 | 25 | 127 |
| Piston | LC45 | 155,5 | 5,15 | 10 | 26 | 125 |
| Piston | LC44 | 165,5 | 4,77 | 8 | 24 | 126 |
| Piston | LC43 | 175,5 | 4,93 | 9 | 22 | 127 |
| Piston | LC42 | 185,5 | 4,71 | 5 | 28 | 126 |
| Piston | LC41 | 195,3 | 5,25 | 11 | 27 | 132 |
| Piston | LC40 | 205,0 | 5,06 | 7 | 25 | 126 |
| Piston | LC39 | 214,8 | 4,80 | 7 | 23 | 126 |
| Piston | LC38 | 224,5 | 4,87 | 4 | 26 | 120 |
| Piston | LC37 | 234,2 | 5,19 | 7 | 25 | 121 |
| Piston | LC36 | 243,9 | 5,18 | 3 | 24 | 122 |
| Piston | LC35 | 253,6 | 5,28 | 5 | 27 | 120 |
| Piston | LC34 | 263,3 | 5,47 | 6 | 26 | 119 |
| Piston | LC33 | 273,0 | 5,29 | 4 | 24 | 119 |
| Piston | LC32 | 282,7 | 5,51 | 6 | 24 | 122 |
| Piston | LC31 | 292,4 | 5,73 | 4 | 25 | 118 |
| Piston | LC30 | 302,1 | 5,29 | 5 | 24 | 121 |
| Piston | LC29 | 311,8 | 5,46 | 1 | 25 | 121 |
| Piston | LC28 | 321,6 | 5,48 | 2 | 25 | 116 |
| Piston | LC27 | 331,3 | 5,74 | 3 | 27 | 117 |
| Piston | LC26 | 341,0 | 5,09 | 1 | 24 | 118 |
| Piston | LC25 | 350,7 | 5,07 | 1 | 22 | 121 |
| Piston | LC24 | 360,4 | 4,91 | 2 | 27 | 121 |
| Piston | LC23 | 370,1 | 5,33 | 1 | 22 | 113 |
| Piston | LC22 | 379,8 | 5,36 | 2 | 24 | 157 |
| Piston | LC21 | 389,5 | 5,28 | 1 | 23 | 120 |
| Piston | LC20 | 399,5 | 6,21 | 1 | 23 | 114 |
| Piston | LC19 | 409,5 | 5,04 | 4 | 25 | 119 |
| Piston | LC18 | 419,5 | 5,82 | 2 | 26 | 120 |
| Piston | LC17 | 429,5 | 5,81 | 3 | 23 | 119 |
| Piston | LC16 | 439,5 | 5,23 | 2 | 26 | 120 |
| Piston | LC15 | 449,5 | 5,83 | 2 | 24 | 117 |
| Piston | LC14 | 459,5 | 5,94 | 3 | 24 | 122 |
| Piston | LC13 | 469,5 | 5,95 | 2 | 23 | 119 |
| Piston | LC12 | 479,5 | 5,69 | 3 | 24 | 119 |
| Piston | LC11 | 489,5 | 5,61 | 1 | 23 | 119 |
| Piston | LC10 | 499,5 | 5,75 | 3 | 25 | 191 |
| Piston | LC9 | 509,5 | 5,56 | 3 | 26 | 123 |
| Piston | LC8 | 519,5 | 5,63 | 1 | 25 | 117 |
| Piston | LC7 | 529,5 | 5,14 | 0 | 25 | 119 |
| Piston | LC6 | 539,5 | 5,25 | 0 | 23 | 117 |
| Piston | LC5 | 549,5 | 5,12 | 2 | 25 | 117 |
| Piston | LC4 | 559,5 | 5,48 | 0 | 26 | 116 |
| Piston | LC3 | 569,5 | 6,03 | 1 | 22 | 121 |
| Piston | LC2 | 579,5 | 5,73 | 0 | 24 | 112 |
| Piston | LC1 | 589,5 | 5,82 | 0 | 24 | 116 |

**Table S5.** ^210^Pb based ages using both the CRS and CIC model (Appleby and Oldfield 1978) for Erstaviken. CRS dates were used to construct the age-depth model for Erstaviken (Fig.4).

| **Core** | **Composite depth (cmbsf)** | **Cumulative mass (g/cm^2^)** | **^210^Pb (dpm/g)** | **^210^Pb**  **cumulative**  **(dpm/g)** | **Age (CRS model)** | **^210^Pb_exc_ (dpm/g)** | **^210^Pb_exc_ (dpm/cm^2^)** | **^210^Pb_exc_ cumulative**  **(dpm)** | **Age (CIC model)** |
| --- | --- | --- | --- | --- | --- | --- | --- | --- | --- |
| Gemini | 0,5 | 0,1 | 28,9 | 307,6 | 2015,0 | 20,6 | 2,4 | 2,4 | 2014,4 |
| Gemini | 2,5 | 0,4 | 26,8 | 278,7 | 2011,8 | 21,5 | 5,3 | 7,7 | 2013,2 |
| Gemini | 3,5 | 0,5 | 25,7 | 251,9 | 2008,6 | 20,6 | 2,8 | 10,6 | 2012,5 |
| Gemini | 5,5 | 0,9 | 18,5 | 226,2 | 2005,1 | 15,3 | 5,8 | 16,4 | 2011,0 |
| Gemini | 10,5 | 2,0 | 17,9 | 207,8 | 2002,4 | 15,0 | 16,9 | 33,2 | 2006,3 |
| Gemini | 15,5 | 3,2 | 14,2 | 189,8 | 1999,5 | 11,1 | 12,7 | 45,9 | 2002,2 |
| Gemini | 20,5 | 4,3 | 15,9 | 175,6 | 1997,0 | 12,5 | 14,3 | 60,2 | 1996,9 |
| Gemini | 25,5 | 5,6 | 13,5 | 159,7 | 1994,0 | 9,8 | 12,4 | 72,6 | 1991,4 |
| Gemini | 30,5 | 6,7 | 12,9 | 146,2 | 1991,1 | 8,9 | 10,3 | 82,9 | 1986,1 |
| Gemini | 34,5 | 7,7 | 14,6 | 133,3 | 1988,2 | 10,3 | 10,0 | 93,0 | 1979,8 |
| Gemini | 35,5 | 7,9 | 14,3 | 118,7 | 1984,4 | 9,9 | 2,1 | 95,1 | 1978,3 |
| Gemini | 36,5 | 8,1 | 13,9 | 104,4 | 1980,3 | 9,7 | 2,0 | 97,1 | 1976,9 |
| Gemini | 37,5 | 8,3 | 12,6 | 90,5 | 1975,7 | 8,7 | 2,1 | 99,2 | 1975,2 |
| Gemini | 38,5 | 8,6 | 12,7 | 77,9 | 1970,9 | 8,6 | 1,9 | 101,1 | 1973,7 |
| Gemini | 39,5 | 8,9 | 12,5 | 65,2 | 1965,2 | 8,3 | 2,5 | 103,6 | 1971,5 |
| Gemini | 40,5 | 9,1 | 12,9 | 52,7 | 1958,4 | 7,8 | 2,1 | 105,7 | 1969,7 |
| Gemini | 45,5 | 10,4 | 11,0 | 39,8 | 1949,3 | 7,4 | 9,1 | 114,8 | 1959,6 |
| Gemini | 50,5 | 11,7 | 9,9 | 28,8 | 1938,9 | 6,5 | 8,4 | 123,2 | 1946,4 |
| Gemini | 55,5 | 13,0 | 9,5 | 18,9 | 1925,5 | 6,2 | 8,3 | 131,5 | 1924,0 |
| Gemini | 60,5 | 14,3 | 9,4 | 9,4 | 1903,0 | 6,5 | 8,2 | 139,7 |  |

**Table S6.** Sample material, sample IDs, ^14^C dates, ^87^Sr/^86^Sr ratios, inferred ^14^C reservoir ages, inferred salinities and calibrated ^14^C ages in years BP, including all 95.45% HPD intervals with the highest probabilities used to construct the age-depth model for Erstaviken (Fig.4).

| **Core** | **Composite depth (cmbsf)** | **Sample material** | **Sample ID** | **^14^C age** | **±1σ** | **^87^Sr/^86^Sr ±1σ** | **±1σ** | **Inferred ^14^C res. age** | **±1σ** | **Inferred salinity** | **±1σ** | **Calibrated ^14^C ages in Cal. yr BP 95.45% HPD intervals (probability)** |  |  |  |
| --- | --- | --- | --- | --- | --- | --- | --- | --- | --- | --- | --- | --- | --- | --- | --- |
| Gemini | 66,5 | *M. Balthica* | LuS12149* | -581 | 30 | 0,70937 | 000075 | 132 | 40 | 6,4 | 0,2 | -7 (see text) |  |  |  |
| Piston | 299,3 | Pine needle | LuS11868 | 1000 | 35 | - | - | - | - | - | - | 973-896 (0.651) | 873-797 (0.304) |  |  |
| Piston | 328,4 | Pine needle | LuS12155 | 1315 | 35 | - | - | - | - | - | - | 1297-1221 (0.697) | 1214-1182 (0.26) |  |  |
| Piston | 403,2 | Leaf fragment | LuS12154 | 2060 | 60 | - | - | - | - | - | - | 2295-2270 (0.021) | 2154-1883 (0.934) |  |  |
| Piston | 425,4 | Pine needle | LuS11867 | 2020 | 40 | - | - | - | - | - | - | 2108-2083 (0.04) | 2065-1884 (0.915) |  |  |
| Piston | 452,5 | Leaf fragment | LuS12153 | 2120 | 35 | - | - | - | - | - | - | 2298-2259 (0.075) | 2157-1995 (0.881) |  |  |
| Piston | 493,3 | Pine needle | LuS12152 | 2755 | 110 | - | - | - | - | - | - | 3210-2704 (0.949) | 2628-2620 (0.002) | 2556-2545 (0.003) |  |
| Piston | 496,3 | *M. Balthica* | LuS11866 | 2630 | 40 | 0,709272 | 000075 | 210 | 41 | 10,2 | 0,5 | 2705-2628 (0.201) | 2620-2552 (0.136) | 2549-2349 (0.617) |  |
| Piston | 546,6 | *M. Balthica* | LuS11865 | 2800 | 40 | 0,709284 | 000075 | 196 | 41 | 9,5 | 0,4 | 2850-2679 (0.692) | 2640-2608 (0.062) | 2600-2492 (0.201) |  |
| Piston | 548,0 | *M. Balthica* | LuS12150 | 2840 | 40 | 0,70928 | 000075 | 201 | 41 | 9,7 | 0,4 | 2876-2698 (0.899) | 2632-2617 (0.014) | 2588-2538 (0.034) | 2527-2513 (0.008) |
| Piston | 569,7 | *M. Balthica* | LuS11864 | 3035 | 40 | 0,709283 | 000075 | 197 | 41 | 9,5 | 0,4 | 3141-3121 (0.021) | 3114-3093 (0.022) | 3079-2837 (0.874) | 2831-2794 (0.038) |
| Piston | 582,0 | *M. Balthica* | LuS11863 | 3040 | 40 | 0,709273 | 000075 | 209 | 41 | 10,2 | 0,5 | 3139-3128 (0.011) | 3108-3094 (0.013) | 3078-2789 (0.931) |  |

* The ^14^C age for LuS-12149 was reported by the lab as FMC 1.075 ± 0.004.
